# Within-host genetic diversity of SARS-CoV-2 lineages in unvaccinated and vaccinated individuals

**DOI:** 10.1038/s41467-023-37468-y

**Published:** 2023-03-31

**Authors:** Haogao Gu, Ahmed Abdul Quadeer, Pavithra Krishnan, Daisy Y. M. Ng, Lydia D. J. Chang, Gigi Y. Z. Liu, Samuel M. S. Cheng, Tommy T. Y. Lam, Malik Peiris, Matthew R. McKay, Leo L. M. Poon

**Affiliations:** 1 School of Public Health, LKS Faculty of Medicine, The University of Hong Kong, Hong Kong SAR, China; 2 Department of Electronic and Computer Engineering, The Hong Kong University of Science and Technology, Hong Kong SAR, China; 3 Centre for Immunology & Infection, Hong Kong Science and Technology Park, Hong Kong SAR, China; 4 Laboratory of Data Discovery for Health, Hong Kong Science and Technology Park, Hong Kong SAR, China; 5 HKU-Pasteur Research Pole, School of Public Health, LKS Faculty of Medicine, The University of Hong Kong, Hong Kong SAR, China; 6 Department of Electrical and Electronic Engineering, University of Melbourne, Parkville, VIC 3010 Australia; 7 Department of Microbiology and Immunology, The Peter Doherty Institute for Infection and Immunity, University of Melbourne, Melbourne, VIC 3000 Australia; 8 Department of Chemical and Biological Engineering, The Hong Kong University of Science and Technology, Hong Kong SAR, China

**Keywords:** SARS-CoV-2, Policy and public health in microbiology, Viral evolution

## Abstract

Viral and host factors can shape SARS-CoV-2 evolution. However, little is known about lineage-specific and vaccination-specific mutations that occur within individuals. Here, we analysed deep sequencing data from 2,820 SARS-CoV-2 respiratory samples with different viral lineages to describe the patterns of within-host diversity under different conditions, including vaccine-breakthrough infections. In unvaccinated individuals, variant of Concern (VOC) Alpha, Delta, and Omicron respiratory samples were found to have higher within-host diversity and were under neutral to purifying selection at the full genome level compared to non-VOC SARS-CoV-2. Breakthrough infections in 2-dose or 3-dose Comirnaty and CoronaVac vaccinated individuals did not increase levels of non-synonymous mutations and did not change the direction of selection pressure. Vaccine-induced antibody or T cell responses did not appear to have significant impact on within-host SARS-CoV-2 sequence diversification. Our findings suggest that vaccination does not increase exploration of SARS-CoV-2 protein sequence space and may not facilitate emergence of viral variants.

## Abstract (web summary)

There is limited data on within-host SARS-CoV-2 genetic diversity and how it is affected by vaccination. The authors analysed intra-host sequence diversity and found that VOCs may have more sequence variations than non-VOCs and that breakthrough infections in vaccinated individuals do not seem to increase non-silent mutations.

## Introduction

The SARS-CoV-2 pandemic continues to spread globally. Despite the vaccination of over 69% of the world population^1^, the risk of SARS-CoV-2 reinfections and breakthrough infections is increasing due to the emergence of new viral variants^2,3^. Multiple variants of concern (VOC) have demonstrated the ability to evade naturally-acquired or vaccine-induced immunity^4–6^. Therefore, it is crucial to investigate the impact of vaccination on the mutational and evolutionary processes of SARS-CoV-2.

Genomic surveillance has been used to trace the transmission and evolution of SARS-CoV-2 mutations at local, regional, and global scales throughout the pandemic^7–9^. However, there is still limited knowledge of how these mutations originate and accumulate within hosts. Within-host mutations can arise through replication errors or RNA damage/editing^10^ and they may be subject to fixation by stochastic (genetic drift) and deterministic (natural selection) processes. We and others have previously found that the SARS-CoV-2 transmission bottleneck between hosts is narrow^8,11–14^, suggesting that only few virions are transferred from the host during transmission. Most of the low-frequency mutations are not transmitted between patients, which constrains the use of intrahost single nucleotide variants (iSNVs) for effective contact tracing^12,15,16^. However, it remains important to investigate the within-host diversity of SARS-CoV-2 to understand host-level evolutionary forces.

Studying SARS-CoV-2 within-host diversity under different conditions may reveal factors that control virus evolution. Host and viral factors can both contribute to within-host diversity. Host factors such as species (animals/humans)^17^, viral shedding time^18^, and immune status^19^ were previously reported to have effects on intrahost SARS-CoV-2 diversity. It was hypothesized that prolonged infections in hosts with distinct immunological backgrounds (e.g., animals or immunocompromised patients) may hasten viral evolution and lead to the emergence of novel variants^17,20^. However, there is limited knowledge about post-vaccination characteristics of within-host selection pressures, which consistently act on the virus during the entire course of breakthrough infection. Besides, viral factors such as different virus lineages may also affect SARS-CoV-2 replication properties. SARS-CoV-2 VOCs have exhibited varying capacities to evade immunity^4,6^ and acquire higher transmissibility^21,22^. However, it is not clear whether different SARS-CoV-2 variants differ in within-host selection pressures.

Here, we analysed 2,820 deep-sequenced SARS-CoV-2 samples collected in Hong Kong (HK) between mid-2020 and 2022. The within-host diversity in SARS-CoV-2 infections from different lineages (VOCs and non-VOCs) and in breakthrough (Delta or Omicron) infections after Comirnaty or CoronaVac vaccination (two or three doses) were studied. Our results provide insights into the variation of within-host diversity, and the mutational patterns and selection pressures acting on viruses.

## Results

### Diversity of within-host mutations in SARS-CoV-2 samples

We analysed SARS-CoV-2 samples from 2,820 different individuals, alongside 29 other samples for quality control of the analysis (see Methods). The samples were collected from June-2020 to September-2022, covering the third to the fifth COVID-19 waves in HK. All samples had $\ge$90% genome coverage (Sequencing depth: >100; Supplementary Fig. 1), with moderate-to-high viral loads (Ct value $\le$28). The median sequencing depth ranged from 380 to 98,214 per sample. The samples belonged to VOCs (B.1.1.7 (20I or Alpha), B.1.617.2.* (21 J or Delta) and BA.2.* (21 M or Omicron)) and non-VOCs (B.1.36.* (20 A), and B.1.1.63 (20B)). The two non-VOC lineages were detected in the third (20B) and fourth (20 A) COVID-19 waves when no COVID-19 vaccine was available for use in HK^8^. For Delta and Omicron infections, samples from unvaccinated infections and breakthrough infections after Comirnaty or CoronaVac (two-dose and three-dose) vaccination were included.

**Fig. 1:**
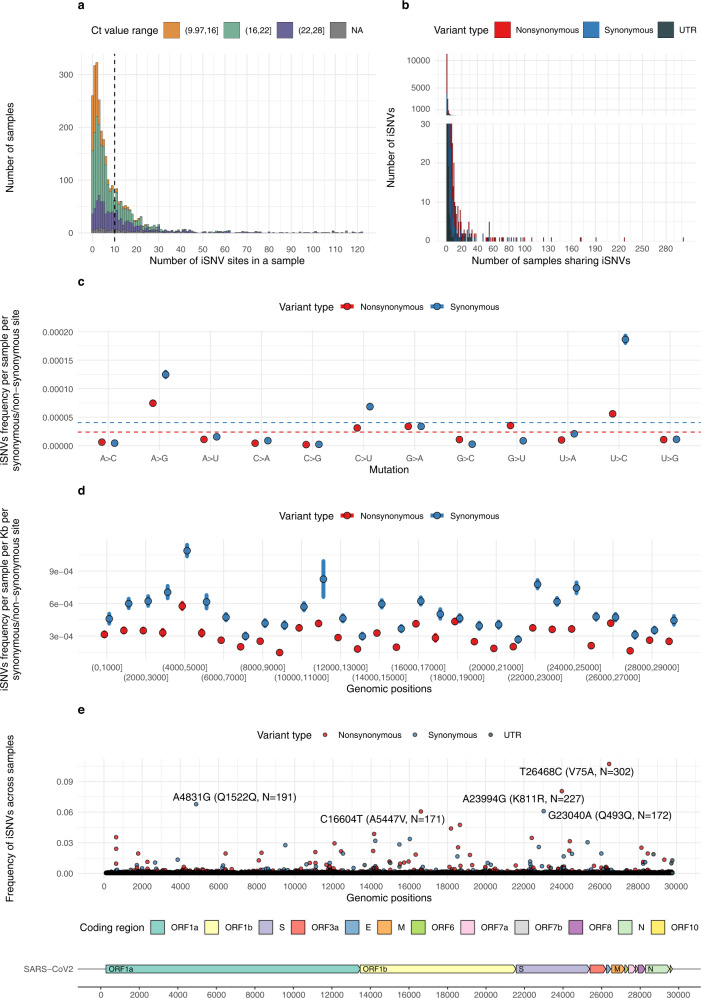
Statistics of within-host mutations in SARS-CoV-2 samples. **a** Distribution of number of iSNV site(s) in each sample, colored by ranges of Ct values. The dashed line shows the mean value of the distribution. **b** Distribution of number of sample(s) sharing iSNVs (e.g., if the iSNV identified in one sample was not shared with any other sample, then the number of samples sharing that iSNV equals to one (x = 1), and so on), colored by variant types, where UTR stands for untranslated region. **c** Distribution of the frequency of iSNVs per sample per synonymous and per non-synonymous site (${d}_{S}$ and ${d}_{N}$) for different types of mutations, colored by variant types. The dashed lines show the average frequency of synonymous and non-synonymous iSNVs among all types of mutations. The points and error bars show mean and standard deviation values based on 10,000 bootstrap replicates at mutation level. **d** Distribution of the frequency of iSNVs per sample per Kb for synonymous and non-synonymous site (${d}_{S}$ per Kb and ${d}_{N}$ per Kb) in different genomic regions of 1Kb length, colored by variant types. The points and error bars show mean and standard deviation values based on 10,000 bootstrap replicates at mutation level. **e** Distribution of high-frequency mutations shared by multiple samples, colored by variant types. Coding regions of the SARS-CoV-2 genome, based on the reference genome (GenBank: MN908947.3), are shown at the bottom of the figure. Source data are provided as a Source Data file.

For determining reliable within-host mutations, quality filtering steps were developed and validated using technical control samples (Methods). We identified 28,353 iSNVs, with allele frequency between 2.5% and 50%, at 13,608 sites from 2560 (90.8%) samples. Of these iSNVs, 17,982 (63.4%) of them were nonsynonymous, 9299 (32.8%) were synonymous, and 172 (3.8%) were in untranslated regions (mutations in the heading and tailing 100 bp regions were excluded). We did not detect iSNVs in the other 260 (9.2%) samples. The mean and median number of iSNVs per sample was 10.05 (Fig. 1a, dashed line) and 5. This iSNV detection rate is higher or lower than previously reported^11,15,16,23^, presumably due to differences in variant filtering criteria. Of the iSNV sites, 11,305 (70.9%) were uniquely observed in single-patient samples. This suggests that most iSNVs are sporadic mutations occurring at distinct positions rather than recurrent mutations occurring at specific hotspots (Fig. 1b).

We found a weak but significant correlation between viral load (Ct) and the number of iSNVs per Kb. Samples with higher viral load (lower Ct) generally had less iSNVs (Fig. 1a and Supplementary Fig. 2A). Similarly, higher viral load also weakly correlated to lower nucleotide diversity (Supplementary Fig. 2C). However, the correlation between detection lag and number of iSNVs was negligible (correlation coefficient R < 0.1, Supplementary Fig. 2E). We also found that the correlation between viral load and minor allele frequency (MAF) was negligible (correlation coefficient R < 0.1, Supplementary Fig. 2D). Consistent with other studies^11,12,15,16^, these results (Supplementary Figs. 2A, C, E) suggest that enrichment of iSNVs negatively correlates with viral load, but varies less with time from symptom onset^16^. Results from our quality control experiments with serially-diluted samples (Methods) suggest that samples with a Ct $\le$28 have good reproducibility in iSNVs detection if appropriate filtering criteria is used. To avoid artefacts due to low viral load^16^, we only included samples with a Ct $\le$28 and adjusted the number of iSNVs per Kb and nucleotide diversity ($\pi$) by linear regression functions (Methods) in the downstream comparative analysis. With these adjustments, all reported correlations (Supplementary Figs. 2A, C, E) became insignificant (Supplementary Fig. 2B, D, F). We found the mean number of iSNVs per Kb to be 0.345 and the highest incidence of iSNVs were found in the spike (S) and envelope (E) genes (Supplementary Table 1).

Consistent with previous reports^23,24^, we found some mutation bias (C→U, G→A, A→G, U→C, and G→U), measured in terms of the number of synonymous/nonsynonymous iSNVs per synonymous/nonsynonymous site (i.e., ${d}_{S}$ and ${d}_{N}$) (Fig. 1c; points above the dashed lines). The high frequency of C→U/G→A and A→G/U→C mutations support the hypothesis of RNA editing via APOlipoprotein B Editing Complex (APOBEC) and Adenosine Deaminase Acting on RNA (ADAR) enzymes^10,25^, respectively. The G→U mutation may relate to Reactive Oxygen Species (ROS)-related processes^26^. We observed uneven ${d}_{S}$ and ${d}_{N}$ (P < 0.001, Kruskal-Wallis rank sum test among regions with length of 1Kb) across the genome and notably a relatively high level of ${d}_{S}$ was found in the spike gene region (genomic position from 22000 to 25000 in Fig. 1d). For all ORFs, the number of synonymous iSNVs per Kb per synonymous site (${d}_{S}\,{{{{{\rm{per\; Kb}}}}}}$) are higher than the average number of nonsynonymous iSNVs per Kb per non-synonymous site (${d}_{N}\,{{{{{\rm{per\; Kb}}}}}}$) (Fig. 1d). There were some shared iSNVs, i.e., found in multiple patients, with five of them (labelled in Fig. 1e) observed in more than 20 samples (frequency>1%). The V75A (E) and K811R (S) mutations were mainly found in the 20 A and 20B lineages, while the Q1522Q (ORF1ab), Q493Q (S) and A5447V (ORF1ab) mutations were mainly found in Omicron and Delta lineages (Supplementary Table 2).

### In unvaccinated individuals, VOC samples exhibit higher within-host diversity than non-VOC samples and are under neutral to purifying selection

To study the within-host diversity between different groups, we calculated the incidence of iSNVs (adjusted number of iSNVs per Kb), abundance of iSNVs (MAF for iSNVs), and nucleotide diversity ($\pi$, adjusted average number of nucleotide differences per site between pairwise reads)^27^ for samples within each group (Methods). Combinational use of the three complementary indices can help illustrate viral mutant spectrum dynamics^28^. Essentially, incidence of iSNVs corresponds to counts of mutational sites in a sample (the breadth of the mutant spectrum), abundance of iSNVs reflects the mutational frequency of each site in a sample (the height/intensity of the mutant spectrum), and nucleotide diversity ($\pi$) represents a functional index based on the total pairwise difference among observed haplotypes (the degree of polymorphism of iSNVs within a sample). Nucleotide diversity ($\pi$) can be further characterized as synonymous and nonsynonymous nucleotide diversity (${\pi }_{S}$ and ${\pi }_{N}$) in coding regions to study the direction of selection (Methods).

**Fig. 2:**
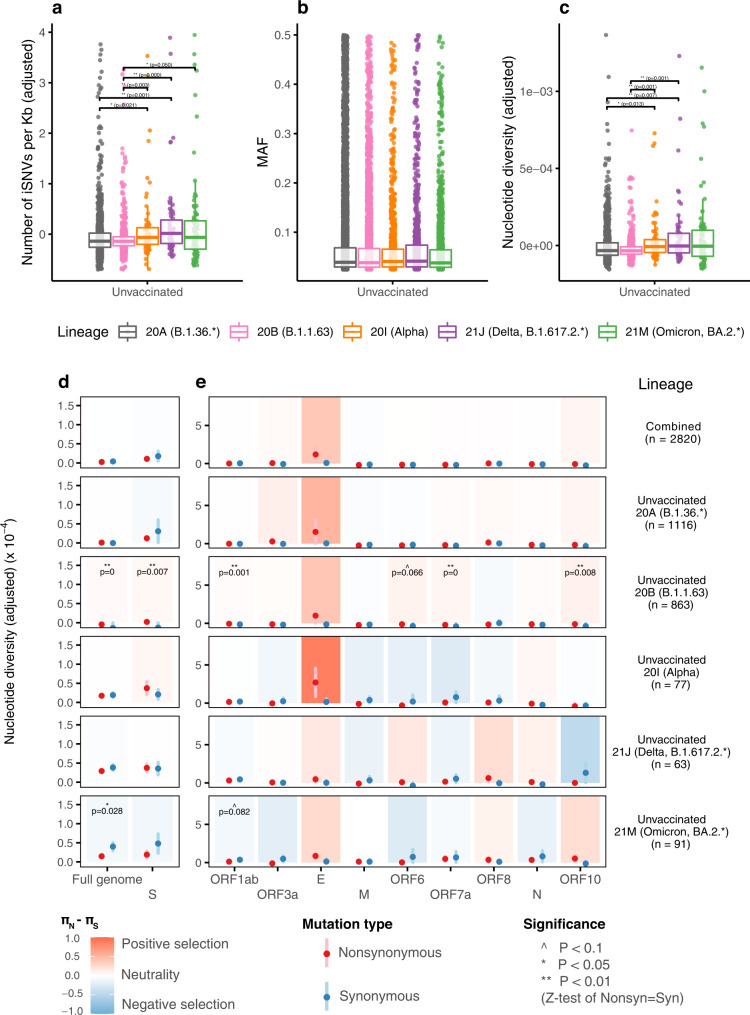
Comparison of within-host mutation profiles between unvaccinated VOC and unvaccinated non-VOC samples. **a–c** Full genome incidence of iSNVs (adjusted number of iSNVs per Kb), abundance of iSNVs (minor allele frequencies, MAF), and adjusted nucleotide diversity (π) of different samples. For all box plots, the bold horizontal line inside the box shows the median, the upper and lower edges of the box indicate the first and the third quartiles, and whiskers extend to span a 1.5 interquartile range from the edges. Pairwise comparisons between groups were performed by two-sided two-sample Wilcoxon tests; the pairs with Benjamini-Hochberg (BH) corrected *P* value $\le 0.01$ and $\le 0.05$ are labelled with “**” and “*” respectively. **d–e** Full-genome and gene-specific within-host nonsynonymous nucleotide diversity (${\pi }_{N}$) and synonymous nucleotide diversity (${\pi }_{S}$) in samples from different groups. The points and error bars show the mean and standard deviation values under 10,000 bootstrap replicates at codon level. Significance was evaluated using two-sided Z-tests of the null hypothesis that ${\pi }_{N}-{\pi }_{S}=0$ (10,000 bootstrap replicates, codon unit); P-value $\le 0.01$, $\le 0.05$ and $\le 0.10\,$are labelled with “**”, “*” and “^”, respectively. The number of biologically independent samples in each group are shown in Supplementary Table 5. Source data are provided as a Source Data file.

Lineage-specific effects on iSNVs were characterized by comparing unvaccinated samples between lineages. We found Alpha and Delta samples without vaccination (designated “unvaccinated Alpha and Delta samples”) all had higher incidence rates of iSNVs than samples from the non-VOC 20 A or 20B lineage (*P* < 0.05; Fig. 2a and Supplementary Table 3). The unvaccinated Omicron samples also had a significantly higher incidence of iSNVs than unvaccinated 20B samples, but the increase in incidence compared to unvaccinated 20 A samples was not statistically significant. Notably, the median incidence of iSNVs of each VOC lineage (Alpha, Delta and Omicron) is higher in all cases than non-VOC lineages (Fig. 2a). No significant difference was observed between the abundance of iSNVs across unvaccinated VOC and non-VOC samples (Fig. 2b). Similar to what was observed for the incidence of iSNVs, the nucleotide diversities between unvaccinated Alpha, Delta and Omicron samples were statistically similar. The Alpha or Delta samples was significantly higher than those from non-VOC lineages (Fig. 2c and Supplementary Table 3). Although the median nucleotide diversity for unvaccinated Omicron samples was higher than non-VOC groups (20 A or 20B), the differences were statistically insignificant. Overall, our data suggests that VOCs tend to induce greater within-host genetic variation than non-VOCs upon infection. But this ability might vary amongst VOCs.

We found the overall SARS-CoV-2 samples in this study were under weak or neutral selection forces (top row, Full genome column in Fig. 2d). The mean value of ${\pi }_{N}-{\pi }_{S}$ is $-0.15\times {10}^{-5}$ (${\pi }_{N}/{\pi }_{S}$ = 0.62) across the full genome for all samples, which agrees with previous reports^11^ (${\pi }_{N}/{\pi }_{S}$ = 0.55), but differs from what was observed in other mammals^17^. For the unvaccinated non-VOC 20 A and 20B samples, neutral to positive (${\pi }_{N}\approx {\pi }_{S}$ or ${\pi }_{N} > {\pi }_{S}$) selection was observed at the full-genome level (Fig. 2d and Supplementary Table 4), and the difference is statistically significant for 20B samples. By contrast, all three unvaccinated VOC samples had overall neutral to purifying selection (${\pi }_{N}\approx {\pi }_{S}$ or ${\pi }_{N} < {\pi }_{S}$) at the full genome level. Notably for the unvaccinated Omicron samples, statistically significant purifying selection was observed, which was mainly due to lowered (comparing to Alpha and Delta samples) level of nonsynonymous nucleotide diversity than synonymous nucleotide diversity (the sixth row, Full genome column in Fig. 2d and Supplementary Table 4). At the individual gene level, statistically significant evidence for positive selection was only observed in unvaccinated 20B samples (regions ORF1ab, spike, ORF6, ORF7a and ORF10, Figs. 2d and e). This result suggests that, unlike viruses from non-VOCs lineages, those from VOC lineages are mostly under neutral selection pressure at the within-host individual gene level.

### Vaccination may affect the within-host mutation diversity but does not induce more non-synonymous mutations

**Fig. 3:**
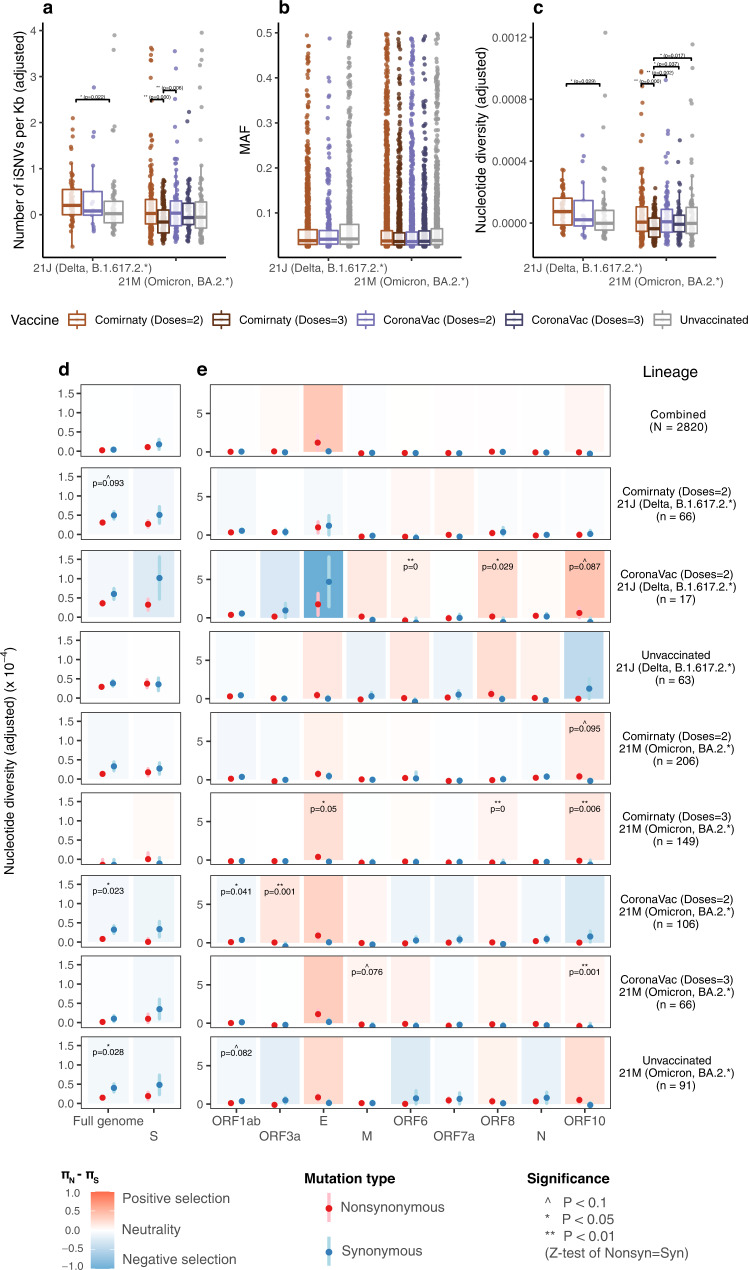
Comparison of within-host mutation profiles between vaccinated and unvaccinated Delta and Omicron samples. **a**–**c** Full-genome incidence of iSNVs (adjusted number of iSNVs per Kb), abundance of iSNVs (minor allele frequencies, MAF) and adjusted nucleotide diversity (π) of different samples. For all box plots, the bold horizontal line inside the box shows the median, the upper and lower edges of the box indicate the first and the third quartiles, and whiskers extend to span a 1.5 interquartile range from the edges. Pairwise comparisons between groups were performed by the two-sided two-sample Wilcoxon test; the pairs with Benjamini-Hochberg (BH) corrected *P* value $\le 0.01$ and $\le 0.05$ are labelled with “**” and “*” respectively. **d**–**e** Full-genome and gene-specific within-host nonsynonymous nucleotide diversity (${\pi }_{N}$) and synonymous nucleotide diversity (${\pi }_{S}$) in samples from different groups. The points and error bars showed the mean and standard deviation values under 10,000 bootstrap replicates at codon level. Significance was evaluated using two-sided Z-tests of the null hypothesis that ${\pi }_{N}-{\pi }_{S}=0$ (10,000 bootstrap replicates, codon unit); P-value $\le 0.01$, $\le 0.05$ and $\le 0.10\,$were labelled with “**”, “*” and “^”, respectively. The number of biologically independent samples in each group are shown in Supplementary Table 5. Source data are provided as a Source Data file.

The incidence of iSNVs and nucleotide diversity may be affected by vaccination. By studying breakthrough infections from vaccinated (with two-doses or three-doses of Comirnaty or CoronaVac vaccines) patients, we found that the incidence of iSNVs in 2-dose Comirnaty Delta samples was significantly higher than that from the unvaccinated Delta samples (Fig. 3a) and the 2-dose Comirnaty Omicron samples (Supplementary Fig. 3A). Within Delta samples, higher incidence of iSNVs in Comirnaty samples compared to unvaccinated samples suggests vaccine-specific effects on within-host mutation rate. However, a similar effect was not observed for Omicron samples (Fig. 3a). One possible explanation for the difference between Delta and Omicron samples could be the waning of vaccine effectiveness, as overall a longer time had passed since receiving the second dose for Omicron-infected vaccinated patients in our data (Supplementary Fig. 4). It is also possible that different levels of immune evasion between Omicron and Delta infections may play a role, since neutralizing antibody titers induced by the Comirnaty vaccine against Omicron were lower than those against Delta^29^. In contrast to Delta samples, Omicron samples from vaccinated individuals did not exhibit increased within-host mutation diversity with 2-dose or 3-dose vaccination. Intriguingly, we found the 3-dose Comirnaty Omicron samples even had a lower level of incidence of iSNVs (Fig. 3a) than the two-dose Comirnaty/CoronaVac vaccinated Omicron samples. The 3-dose Comirnaty samples also had the lowest level of nucleotide diversities among groups (Fig. 3c). Consistent with high incidence of iSNVs, the nucleotide diversity of 2-dose Comirnaty Delta samples was higher than that from the unvaccinated Delta samples (Fig. 3c). Together, these results suggest that vaccination may increase the within-host mutation diversity in Delta samples, but not in Omicron samples. Three-dose Comirnaty vaccination may even be associated with decreased within-host mutation diversity in Omicron samples.

Neutral to purifying selection was observed in all vaccinated groups at the full-genome level (column Full genome in Fig. 3d). In 2-dose Comirnaty Delta samples, marginally significant purifying selection pressure (${\pi }_{N}$ < ${\pi }_{S}$) at the full-genome level were observed (second row, column Full genome in Fig. 3d). The significant purifying selection in 2-dose Comirnaty Delta samples was mainly contributed by increased ${\pi }_{S}$ (${\pi }_{S}$ = 4.97 and 3.84 for 2-dose Comirnaty Delta and unvaccinated Delta samples respectively, Supplementary Table 4). Both the ${\pi }_{N}$ and ${\pi }_{S}$ decreased in 3-dose vaccinated Omicron groups (Fig. 3d), in line with the lower level of incidence of iSNVs and nucleotide diversity observed in 3-dose Comirnaty/CoronaVac vaccinated Omicron samples (Fig. 3a and c). At the spike gene level, all the vaccinated or unvaccinated VOC groups are under neutral selection pressure. Collectively, although vaccination may increase (decrease) the within-host diversity in Delta (Omicron) samples as mentioned above, we did not observe significant change in the direction of selection pressures by vaccination. Neutral and purifying selection were predominant at the full genome level, with possible positive selection sporadically observed in genes ORF3a, E, ORF6, ORF8 and ORF10 in some vaccinated groups (Fig. 3e).

Positive selection in coding regions of VOC-specific and vaccination-specific samples (Figs. 2e and 3e) suggests diversifying mutations that can potentially lead to higher chance of phenotypic changes. To identify putative hotspot regions with excessive positive selection, we analysed sliding windows (size of 30 codons) across each protein-coding region. Consistent to the results above, we found most genomic regions were under purifying selection (Supplementary Fig. 5).

### No significant selection on within-host mutations from immune pressure

To investigate whether the within-host mutations detected in our vaccinated samples enable immune escape, we studied the overlaps of identified within-host mutations with known neutralizing antibody (nAb) escape mutations in the spike gene and with experimentally-determined T cell epitopes across the full genome.

**Fig. 4:**
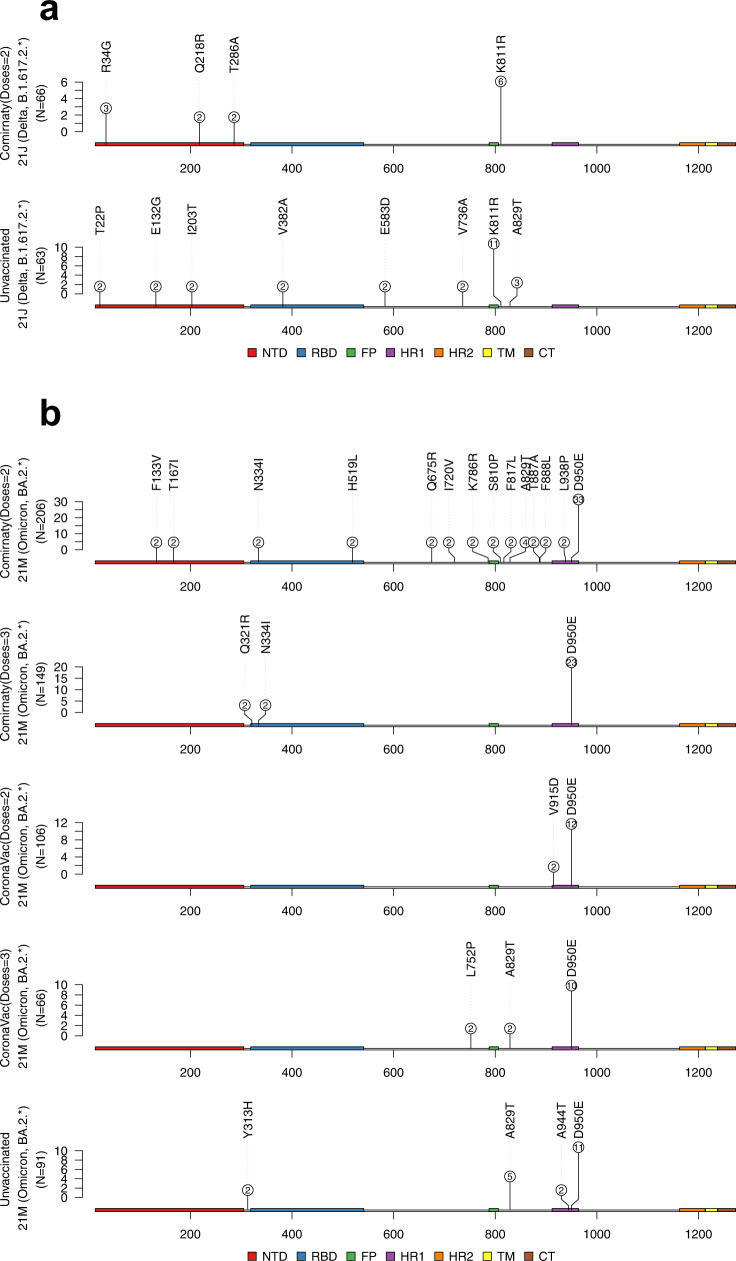
Recurrent spike mutations identified in unvaccinated and vaccinated Delta and Omicron samples. The numbers in each circle represent the number of mutations identified in samples in respective groups. **a** Identified within-host mutations in Delta samples (the data for group CoronaVac (Doses=2) 21 J (Delta, B.1.617.2.*) is not available as no recurrent mutation was found in this group). **b** Identified within-host mutations in Omicron samples. Source data are provided as a Source Data file.

Our identified mutations did not significantly cluster in any specific regions of the spike gene (Figs. 4a and b). We observed one recurrent RBD mutation in unvaccinated Delta samples (V382A, Fig. 4a) and three different recurrent RBD mutations in Comirnaty Omicron samples (Q321R, N334I and H519L, Fig. 4b). Except for the N334I mutation found in four different Comirnaty Omicron samples (Fig. 4b), which may have mild effects on antibody escape (3.1% of studied nAb predicted to escape; Methods), the other identified recurrent mutations in the RBD region in all Omicron and Delta samples have little impact to antibody escape. For the NTD region, none of the recurrent within-host mutations in vaccinated/unvaccinated Omicron and Delta samples locates at the NTD-antigenic supersite^30^ or affects NTD-targeting nAbs-mediated neturalization^31,32^. We noticed a highly recurring HR1 mutation D950E in Omicron samples (Fig. 4B), its function is not clear but previous reports suggest that a D950N may contribute to the regulation of spike protein dynamics^33^.

In addition to nAbs escape mutations, T cell escape mutants have been shown to be selected under immune pressure in influenza infection^34,35^. However, the relationship between within-host mutations and T-cell responses induced by SARS-CoV-2 infection or vaccination remains largely unknown. To investigate whether iSNVs found in breakthrough infections are related to host T cell responses, we studied the overlap between observed iSNVs and known T cell epitopes. A total of 1,802 CD8^+^-specific and 1,058 CD4^+^-specific T cell epitope-HLA (human leukocyte antigen) pairs were compiled (Methods). The distributions of these epitope-HLA pairs across SARS-CoV-2 proteins and across HLAs are shown in Supplementary Fig. 6. Considering T cell epitopes across all proteins, the average number of overlapping CD8^+^ and CD4^+^ epitopes permutation was generally similar between different groups (Supplementary Fig. 7).

**Fig. 5:**
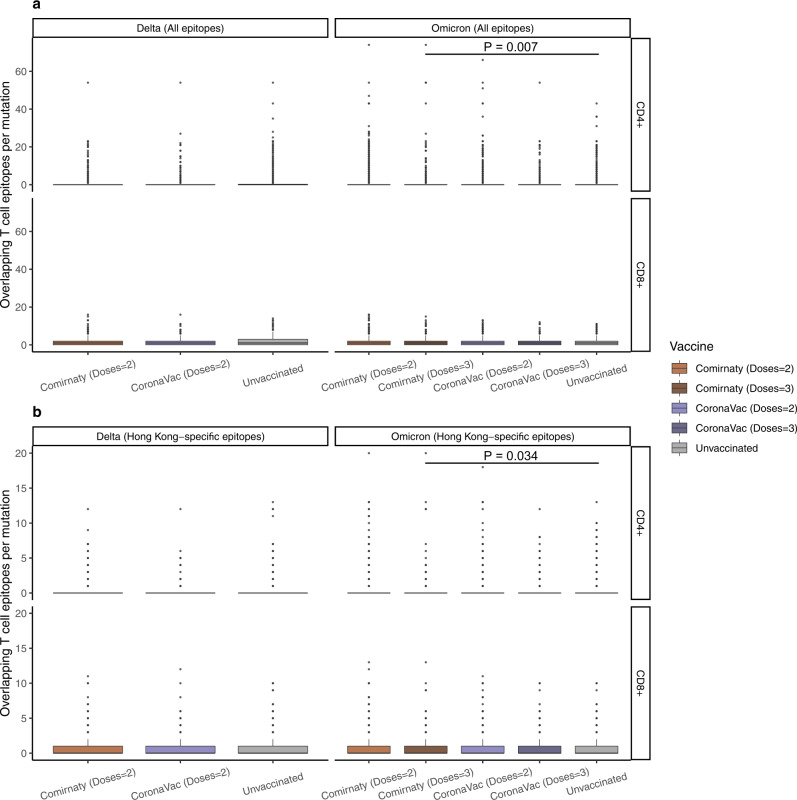
Overlapping known SARS-CoV-2 CD8^+^ and CD4^+^ T cell epitopes per mutation in unvaccinated and vaccinated Delta and Omicron samples. **a** Analysis based on all known SARS-CoV-2 T cell epitopes. **b** Analysis based on T cell epitopes associated with HLA alleles prevalent in the Hong Kong population. Pairwise comparisons within groups were performed by the two-sided two-sample Wilcoxon test. For all box plots, the bold horizontal line inside the box shows the median, the upper and lower edges of the box indicate the first and the third quartiles, and whiskers extend to span a 1.5 interquartile range from the edges. The sample size of data in each group are shown in Supplementary Fig. 6 and 9. Source data are provided as a Source Data file.

For the CD4^+^ T cell epitopes, we observed significantly less overlap per mutation for 3-dose Comirnaty Omicron samples compared to the corresponding unvaccinated samples (Fig. 5a; top panel). A similar trend was observed for CD8^+^ T cell epitopes (Fig. 5a; bottom panel), although the result was not statistically significant (*P* = 0.1). These results are consistent with the lower incidence of iSNVs and nucleotide diversity observed for 3-dose Comirnaty Omicron samples compared to the respective unvaccinated samples (Fig. 3a). Overall, our analysis suggests that 3-dose Comirnaty vaccination may be potentially limiting T cell escape mutations in within-host viral evolution. When limited to mutations within the spike gene, no significant difference was observed between the overlap per mutation with T cell epitopes for any group (Supplementary Fig. 8A).

Since the samples were sequenced from HK cases, we repeated the above analysis while focusing on the epitopes associated with HLAs prevalent in the HK population (Supplementary Fig. 9, Methods). We only observed that the 3-dose Comirnaty Omicron group has significantly less overlapping CD4^+^ T cell epitopes per mutation than the corresponding unvaccinated group at the full genome level (Fig. 5b). More generally, similar to the above results for mutations in the spike gene (Supplementary Fig. 8A), we observed no significant difference among groups in this case as well (Supplementary Fig. 8B). Performing a similar analysis at the individual HLA allele level also did not reveal significant difference between vaccinated and unvaccinated groups for nearly all HLA alleles (Supplementary Fig. 10).

Overall, we did not identify a surge of antibody escape mutations in any group, and different groups had a similar level of mutation rates in T cell epitope regions, except for a lower rate observed among 3-dose Comirnaty Omicron samples compared to unvaccinated samples.

## Discussion

In this study, we have analysed 2,820 SARS-CoV-2 samples to estimate the intrahost variation of SARS-CoV-2 under different conditions. Similar to earlier studies, we show that the incidence of iSNVs in SARS-CoV-2 samples is low (median iSNVs per sample: 5) and that sample viral loads negatively correlate with within-host mutation rates^11,12,15,16^, which suggests low viral load specimens are prone to bias toward falsely high iSNVs rates. In agreement with reports from Tonkin-Hill et al^15^. where SARS-CoV-2 samples with lower Ct value show good concordance in allele frequencies between replicates, we also found the cut-off of Ct $\le$28 can be used in combination of proper sequencing depth and MAF cut-offs to ensure reproducibility in iSNVs calling. Evidence of RNA editing at the full genome level, e.g., the widely reported biased C→U/G→A and A→G/U→C pairs of mutations^23,24^, was observed in our study. We also found strand asymmetry of G→U mutations in our data (higher frequency of G→U than C→A), which are suggestive of RNA damage or RNA editing (rather than replication errors) on the plus stand^15^ and possible association with ROS-related processes^26^. The frequency of synonymous mutations is higher than expected (${d}_{N} < \,{d}_{S}$). Collectively, the general within-host virus sequence diversity in the samples from HK was comparable to those observed from the ancestral SARS-CoV-2^11,16^.

Different lineages of SARS-CoV-2 have different properties, including different levels of transmissibility^21,22^, disease severity^36,37^, viral load^37,38^, tissue affinity^39^, ability of vaccine breakthrough^4–6^, etc. Here, we found SARS-CoV-2 VOC Alpha, Delta and Omicron from unvaccinated individuals may have higher within-host mutation rate and/or nucleotide diversity than non-VOC lineages. Such increased mutation rate is independent of viral load, potentially due to differing intrinsic biological properties between variants. As the entire infected population in HK by the end of 2021 was <0.2%, our observation is unlikely affected by interference induced by prior natural infection. Various mutations have been shown to account for different viral properties, e.g., ACE2 binding (e.g., K417N, N501Y)^40^, and immune escape (e.g., T478K, L452R)^41^. The increased nonsynonymous nucleotide diversity in VOC samples (Supplementary Table 4) suggest that VOC have a greater capacity to explore protein sequence space and therefore are more likely to incur a fitness change. This result is in line with VOCs’ ability to spread and result in multiple sublineages, and warrants close monitoring of their molecular evolution in the future. Specifically for Omicron samples, we observed lower level of non-synonymous mutations compared to Alpha and Delta samples, thus resulting in a significant purifying selection at the full genome level. This result is in line with a recent study, which shows that while the rate of synonymous mutation was stable overtime, the rate of non-synonymous mutation was initially high but dropped significantly in 2022^42^. Interestingly, although some functional viral mutations (particularly those in the spike gene) were selected along the evolution of SARS-CoV-2 (a long-term positive selection), both the population-based^42^ and intra-host (this study) data suggest an overall short-term neutral/purifying selection on the whole viral genome. Our result at the within-host level, together with the above findings at the population level, suggest possible adaptive evolution of SARS-CoV-2. Nonetheless, it is essential for others to use different within-host SARS-CoV-2 sequencing datasets to validate our findings.

Vaccination is another factor which may affect the within-host virus evolution. We studied samples from Comirnaty and CoronaVac vaccine breakthrough infections and found that vaccination may be associated with changed mutation rates but might not change selection pressure. We found 2-dose Comirnaty vaccination was associated with increased synonymous nucleotide diversity and marginally significant purifying selection pressure at the full genome level, while similar effects of 2-dose CoronaVac vaccination was not as significant. Notably, the increased nucleotide diversity in specimens of Delta breakthrough infection in 2-dose Comirnaty vaccinated individuals is mostly synonymous rather than non-synonymous (Supplementary Table 4). Comirnaty vaccine is known to be more immunogenic than CoronaVac vaccine^43^ and this may contribute to our observation. It is also relevant to note that Comirnaty vaccine only has the spike protein as an immunogen but appears to impact on purifying selection elsewhere in the genome. Crucially, vaccination does not increase exploration of the protein sequence space as non-synonymous nucleotide diversity does not seem to be increasing. For Omicron virus samples, we did not observe significant changes in incidence of iSNVs, nucleotide diversity or selection pressure in samples with 2-dose Comirnaty/CoronaVac vaccination. However, 3-dose Comirnaty vaccination virus samples seemed to have significantly lower incidence of iSNVs and nucleotide diversity than 2-dose or unvaccinated Omicron virus samples. The viral loads of 3-dose samples are similar to viral loads of other samples (Supplementary Fig. 13), suggesting that vaccination did not directly suppress viral replication, but might have limited exploration of sequence space. The low diversity observed in 3-dose Comirnaty Omicron samples was not as significant in 3-dose CoronaVac Omicron samples (there was no significant difference between collection lags or time since last dose between the two vaccines), possibly due to immunogenic difference between the two vaccines. We, however, do not exclude alternative hypotheses to explain this observation. Further investigations on this topic are warranted. Overall, 2-dose Comirnaty vaccination seemingly amplifies the within-host mutations in Delta virus samples, but 3-dose Comirnaty vaccination reduces the within-host mutations in Omicron samples. All the vaccinated groups did not have higher level of non-synonymous mutations.

As HK used an elimination strategy to control COVID-19, the individuals investigated in our study can be reliably categorised as immunologically naïve or vaccinated individuals, which is a significant advantage of our study. Nonetheless, our study has several limitations. Most of the studied cases have only single-timepoint samples, making it hard to study the temporal changes of within-host selection pressures. Although we made an effort to account for biases from sampling and different viral loads, false positive variant calls can still be an issue in analysing next-generation sequencing data, particularly from clinical samples that are of limited availability. A well-planned cohort, which can control major potential confounding factors, e.g., different demographic backgrounds, vaccination time lags, sample collection time points, and sequencing conditions, would provide a more robust estimation on iSNVs profiles in SARS-CoV-2 infections. Besides, in studying the effect of T cell pressure on within-host viral evolution, we could not perform an individual-based analysis since HLA typing of the patients was not possible in our study. In addition, since most of the individuals in our study were either infection naïve or vaccinated prior to infection, the effect of hybrid immunity on SARS-CoV-2 within-host evolution could not be addressed and requires further investigation.

In conclusion, our work suggests that SARS-CoV-2 within-host evolution may exhibit different patterns in different virus lineages and in vaccinated individuals. We found that 2-dose or 3-dose Comirnaty and CoronaVac COVID-19 vaccination does not seem to increase non-synonymous mutations in VOCs, suggesting that vaccination may limit the exploration of protein sequence space and the emergence of more viral variants.

## Methods

### Samples, patient characteristics and sequencing

This study was conducted under ethical approval from the Institutional Review Board of the University of Hong Kong (UW 20-168). Written informed consent was waived, as the study used deidentified, archived samples from citywide public health screening programs. We included Illumina amplicon data from 2,820 samples from lineages B.1.1.7 (20I or Alpha), B.1.617.2.* (21 J or Delta), BA.2.* (21 M or Omicron), B.1.36.* (20 A), and B.1.1.63 (20B) (variants in the third and fourth local wave) collected from 2020-06-24 to 2022-09-15 in HK. These were archived SARS-CoV-2 samples confirmed by citywide public health screening programs. The dataset includes samples from 2,820 individuals, where 1400 are male, 1413 are female and 7 are not available. The mean and median age of the studied population are 45.23 and 45 respectively, details are disclosed in the anonymised metadata. All the samples were from patients who were either unvaccinated or at least fully vaccinated (received two or three doses of vaccines) with Comirnaty or CoronaVac vaccines. To minimise potential bias from re-infection, only Hong Kong local cases were included for the Omicron samples (the re-infection rate for Hong Kong population by the end of 2021 was <0.2%, due to the elimination strategy applied in HK). The number of samples included in the analysis are presented in Supplementary Table 5. The metadata and vaccination records of RT-PCR-confirmed cases of COVID-19 were collected from the public data released by HK government since July 29, 2021 (https://gia.info.gov.hk/general/202107/29/P2021072900356_373472_1_1627542548101.pdf).

The respiratory samples were mainly from throat saliva, throat and nasal swab, nasal swab, nasopharyngeal swab, nasopharyngeal aspirate etc. Our analysis showed that the incidences of iSNVs (Ct value adjusted) are not significantly different between specimen types (Supplementary Fig. 11). Above 90% of the studied samples were collected within 5 days after symptom onset (median: 2 days, mean: 2.3 days, for symptomatic cases). We did not find significant difference in the collection lag between groups, except that the unvaccinated 20B group had longer collection lag than the unvaccinated Delta group with median difference of 1 day (Supplementary Fig. 12). The Ct values are similar between groups except for the 20B group which had higher viral loads (Supplementary Fig. 13). Subsequent analysis suggests that the cause of this observed higher viral load may be the significant higher average age in patients from the HK third wave (20B group), as we found higher age weakly correlates with lower Ct values (Supplementary Fig. 14A). However, the higher age in the 20B group is not likely to bias the analysis of iSNVs since the correlation between age and incidence of iSNVs (Ct value adjusted) are not significant (Supplementary Fig. 14B). Overall, these tests suggest that the analysis of iSNVs in our work across different lineages and vaccination statuses is not influenced by sampling or patient bias.

Respiratory samples were sent to a World Health Organization reference laboratory at the University of HK for full-genome analyses (Institutional Review Board no. UW 20–168). RNA was extracted using QIAamp Viral RNA Mini Kit (Qiagen, Cat. No.: 52906). Extracted RNA were reverse transcribed using with multiple gene-specific in-house primers (https://github.com/Leo-Poon-Lab/mutations-under-sarscov2-vaccination/blob/main/Source%20Data/SARS-CoV-2%20full%20genome%20primers%20HKU.xlsx) targeting different regions of the viral genome. The synthesized cDNA was then subjected to multiple overlapping 2-kb PCRs for full-genome amplification using LA Taq DNA polymerase (Takara, Cat. No.: RR002M). PCR amplicons were purified using QIAquick PCR Purification kit (Qiagen, Cat. No.: 28106). Purified amplicons obtained from the same specimen were pooled for library preparation using DNA Prep (Illumina, Cat. No.: 20018704). Libraries were then quantified using Qubit dsDNA HS Assay Kits (Life Technologies, Cat. No.: Q32851) and sequenced in Novaseq or iSeq100 sequencer (Illumina) according to manufacturer’s guideline. Generated sequencing reads were quality trimmed by fastp with parameters (“-q 30 -5 -3 -c --detect_adapter_for_pe -l 50”). Potential PCR duplicates were removed by samtools markdup (v1.11). The trimmed reads were mapped to a reference virus genome by using BWA-MEM2 (v2.0pre2), and genome consensus was generated by using iVar (v1.3.1) with the PCR primer trimming protocol (minimum sequence depth of 100 and minimum Qvalue of 30).

### Analysis of quality control samples

We included technical control samples for quality control in this study, including 17 manually generated serially-diluted samples (to estimate reproducibility of iSNVs under different viral loads, and to determine proper cut-offs for calling iSNVs), and 12 longitudinal samples from 6 cases (two samples for each case, to study the changes in iSNVs during the course of infection).

#### Determining thresholds of Ct value, sequencing depth and MAF in variant calling

The serially-diluted samples were generated based on an in-house virus cell-culture isolate. The original cell-culture sample (e-1) was diluted in serial (with steps of 10-times concentration difference) for 10 times, resulting in the e-2 to e-11 samples. For e-6 to e-11 samples, the Ct values were greater than 24 and we sequenced them in duplicates (e.g., e-6-1 and e-6-2 duplicates) to avoid outlying results.

Generally, we found samples with higher Ct values had lower sequencing depth across the genome and lower overall genome coverage (Supplementary Fig. 15A and B). We found the results of consensus bases calling agreed among all samples for all bases with sequencing depth > =5, suggesting sequencing accuracy at consensus are high for all samples. To determine the sequencing accuracy at the level of iSNVs, we used the consensus iSNVs (shared by at least two samples among e-1 to e-3 samples) as the true positives, and tested whether they can be detected in the samples with lower viral loads. Based on ROC curves, we found increasing the depth cut-off from 10 to 100 significantly decreased the false positive fraction, independent of different MAF cut-offs (Supplementary Fig. 16). We tested 20 MAF thresholds between 0.005 and 0.100 with step of 0.005, and found that an MAF threshold between 0.015 and 0.030 (median and mode value of 0.025) is optimal for sensitivity and specificity when using a depth cut-off of 100 reads (Supplementary Table 6). Specifically, an MAF threshold of 0.02 is optimal in samples e-1, e-2, e-7, and an MAF threshold of 0.025 is optimal in samples e−5, e-6-1, and e-6-2 (obtaining maximum aera under curves).

From our analysis, we found samples with Ct value >28 generally had poor genome coverage and low true positive fraction in iSNVs detection. Thus, such samples are not suitable for inclusion in the analysis. We also found that a sequencing depth cut-off of 100 reads with MAF threshold of 0.025 performed the best in iSNVs detection by balancing true positive and false positive fractions (more than 80% of the iSNVs can be repeatedly detected in samples with Ct value <28, Supplementary Fig. 15C).

#### Longitudinal samples

During the study period, we obtained 6 pairs of high-quality (CT value <28) longitudinal samples from 6 cases (totalling 12 samples). Among these 6 cases, 5 were infected by a Delta variant (3 vaccinated with 2 doses and 2 unvaccinated), the other one was infected by an Omicron BA.1 variant (vaccinated with 2 doses). Theses samples were all collected within the first three days of patients’ onset of disease (not from prolonged infection, 5 pairs of samples were collected in day-0 and day-1, the other pair of samples was collected in day-0 and day−3). In 5 of the 6 cases, we found the sample viral loads increased in the second timepoint, suggesting a progress of disease. We found that iSNVs were rarely shared between the analysed longitudinal samples (only 1 shared iSNV was detected in 1 pair of samples), but the consensus sequences are highly consistent (median 1.5 nucleotide changes per pair of samples). We did not observe significant different pattern between vaccinated and unvaccinated cases. These results suggest that the iSNVs spectrum may be changing rapidly during the course of infection within individuals, which agrees with recent results observed in normal (non-immunocompromised) patient from others^23,44^.

### Variant calling

To obtain high quality sequencing results, we only included samples with a Ct value $\le$28 and with sufficient sequencing depth and genome coverage (sequencing depth $\ge$100 properly paired reads were required at > =90% of the genomic sites for every sample) after pre-processing (i.e., the above-mentioned reference-based alignment of NGS reads).

The consensus-level single nucleotide polymorphisms (SNPs) and intrahost single nucleotide variants (iSNVs) were determined based on the nucleotide composition of every genomic position (mpileup files from samtools v1.1) with reference to the Wuhan-Hu-01 sequence. To limit the analysis to high quality SNPs and iSNVs, the following filtering criteria were applied:

1. SNVs were called from samtools mpileup files from quality-filtered reads alignment bam files using pysamstats (v1.1.2).
2. After filtering based on MAF threshold of 0.025, we identified 116,193 iSNVs in 2820 samples.
3. After filtering heading/tailing 100 bp UTR region, binding regions of PCR primers, and previously known problematic sites^8^, we identified 85,428 iSNVs in 2820 samples.
4. After filtering for iSNVs by minimum depth of 100 reads, we identified 60,170 iSNVs in 2820 samples.
5. After filtering out iSNVs of structural variants, we identified 42,667 in 2783 samples.
6. After filtering for iSNVs with strong strand bias (we kept iSNVs with strand ratio <1/10), we identified 34,521 iSNVs in 2587 samples.
7. After filtering for serial adjacent disjoint mutations ($\ge$3 mutations within 30 nucleotides sliding window, likely relating to sequencing errors), we identified 32,948 iSNVs in 2586 samples.
8. Finally, removing samples with possible co-infection/contamination, we identified 28,353 iSNVs in 2560 samples.

### Mutation summary statistics

#### Incidence of iSNVs and minor allele frequency

The incidence of iSNVs (number of iSNVs per Kb) was calculated by dividing the number of iSNVs with the number of genomic positions with sufficient coverage of reads (sequencing depth $\ge$ 100). The minor allele frequency (MAF), representing the abundance of iSNVs, was calculated directly from the alignment mpileup files using pysamstats (v1.1.2).

#### Nucleotide diversity (${{{{{\boldsymbol{\pi }}}}}}$)

Nucleotide diversity ($\pi$) is a summary metric of the degree of polymorphism of iSNVs within a sample and is tolerant of biases from sequencing depth^45^. We use it to measure the degree of iSNVs polymorphism within a sample. For every sample, where ${n}_{i}$ sequences (NGS reads) of nucleotide $i$ are observed, nucleotide diversity ($\pi$) can be calculated based on pairwise difference between sequencing reads. as

$$\pi=\,\frac{{\sum }_{i\ne j}{n}_{i}{n}_{j}}{\frac{1}{2}N(N-1)}\,,$$

where *N* is the total number of sequences. In all statistical analysis in this study involving nucleotide diversity, we refer to the Ct value adjusted nucleotide diversity (see below).

#### Adjusting Incidence of iSNVs and nucleotide diversity (${{{{{\boldsymbol{\pi }}}}}}$) by Ct values

The adjusted incidence of iSNVs is the residual (that is response minus fitted values) calculated by a least-squares linear model (“lm” function in R 4.1.0) with the numbers of iSNVs per Kb (response variable) and Ct values (explanatory variable) from all the studied samples. For adjusted nucleotide diversity (${{{{{\rm{\pi }}}}}}$), the linear model was fitted for the mean number of pairwise nucleotide comparisons at the codon level, separately for synonymous and non-synonymous mutations (the numerator of ${\pi }_{S}$ and ${\pi }_{N}$, respectively). The denominator of ${\pi }_{S}$ and ${\pi }_{N}$ was fixed given the SARS-CoV-2 genome; thus, it did not need to be adjusted. The total adjusted nucleotide diversity (π) was calculated by summing the adjusted ${\pi }_{S}$ and ${\pi }_{N}$. Detailed implementation can be found in the provided source code.

### Selection analysis

The nucleotide diversity can be separately calculated for adjusted synonymous (${\pi }_{S}$) and non-synonymous changes (${\pi }_{N}$) in coding regions. We calculated the ${\pi }_{N}$ and ${\pi }_{S}$ in this study using SNPGenie^46^ with self-curated input vcf files based on the above identified iSNVs. For hypothesis testing of selection neutrality (${\pi }_{N}$ = ${\pi }_{S}$), Z-tests using a bootstrap method (codon unit, 10,000 replicates for genes and sliding windows) was applied. The scripts of sliding window analysis for positive selection are largely based on a previous analysis developed by the author of the software (https://github.com/krisp-kwazulu-natal/within-host-diversity-manuscript-analysis-code/blob/a276286680de3723e2b1e70f7a060750892cf8af/scripts/diversity_selection_analyses.R).

### Neutralizing antibody escape mutations

The recurrent spike RBD mutations found in all Omicron and Delta samples were analyzed separately with the Escape Calculator for SARS-CoV-2 RBD^47^. The calculations are based on deep mutational scanning of a large set of RBD targeting antibodies which are known to neutralize the ancestral Wuhan-Hu-1 strain. The mutation escape strength in the Escape Calculator was set to the default value of 2.

For NTD, an antigenic supersite has been defined in McCallum et al.^30^ that is recognised by a large number of NTD-targeting nAbs. It includes the spike regions: 14–20, 140–158 and 245–264. Multiple other NTD mutations have been reported to affect neutralization of NTD-targeting nAbs. These NTD mutations include^31^ A67V, del69-70, T95I, G142D, del143-145, N211I, del212, and ins214 EPE. In ref. ^32^, NTD mutations with strong (del144, R246A), moderate (L18F, T19A, H164Y, D253G, D253Y), and mild (D80A, N149Q, S252F) effect on antibody neutralization were described. This data was collectively used in the overlap analysis of spike NTD mutations (Fig. 4).

### Acquisition of SARS-CoV-2 CD8^+^ and CD4^+^ T cell epitopes

We obtained SARS-CoV-2 CD8^+^ and CD4^+^ T cell epitope data from the dashboard reported by us^48^ (https://www.mckayspcb.com/SARS2TcellEpitopes/; accessed on 15 November 2022) and the Immune Epitope Database (IEDB)^49^ (https://www.iedb.org; accessed on 15 November 2022) by querying for the organism name: “SARS-CoV2” (taxonomy ID: 2697049), host: “human”, and assay: “T cell positive”. The compiled data was processed to only include epitopes with lengths between 9-11 residues for CD8^+^ and 13-20 residues for CD4^+^, which represent the typical range of HLA class I and II epitopes. Removing the epitopes with no or incomplete HLA allele information resulted in a total of 1,802 unique CD8^+^ and 1,058 unique CD4^+^ epitope-HLA pairs (Supplementary Fig. 6). The analysis in Fig. 5A is based on this set of known SARS-CoV-2 T cell epitopes.

For the analysis focused on epitopes targeted by T cells in the HK population (Fig. 5B), we determined class I and class II HLA alleles prevalent in HK. For class I alleles, we employed the IEDB’s “Population Coverage” tool (http://tools.iedb.org/population/) to identify 12 HLA class I alleles that together cover >99% of the HK population (Supplementary Fig. 9A, left panel). A total of 932 unique SARS-CoV-2 CD8^+^ T cell epitopes were associated with these alleles (Supplementary Fig. 9A, right panel). For class II alleles, we employed the Allele Frequency Net Database^50^ (http://www.allelefrequencies.net; accessed on 15 May 2022) and identified 13 HLA class II alleles that have an individual estimated population coverage of >5% in the HK population (Supplementary Fig. 9B, left panel). A total of 307 unique SARS-CoV-2 CD4^+^ T cell epitopes were associated with these alleles (Supplementary Fig. 9B, right panel).

### Overlapping T cell epitopes per mutation

To study whether the within-host mutations (minor allele variants) may affect the T cell response generated against different SARS-CoV-2 lineages and under different vaccination status, we used the metric *Overlapping T cell epitopes per mutation*. It is computed as the number of T cell epitopes overlapping the within-host mutations observed in each group divided by the total number of within-host mutations observed in that group. The T cell epitope data used in the calculation of this metric was either from the complete set (Fig. 5a) or from the set specific to the HK population (Fig. 5b).

### Statistical analysis

For bootstrapping analysis, the measurement can be taken from the same sample measured repeatedly. For the other tests (e.g., Wilcoxon tests), the measurements were taken from distinct samples. All the statistical tests in this study are two-sided and no adjustment for multiple comparisons was performed unless specified.

### Reporting summary

Further information on research design is available in the Nature Portfolio Reporting Summary linked to this article.

### Supplementary information

Supplementary Information

Peer Review File

Reporting Summary

### Source data

Source Data

## Data Availability

The sequencing data generated in this study have been deposited in the NCBI Sequence Read Archive (SRA) under Bioproject accession code PRJNA930974. The processed anonymised metadata are deposited at Github (https://github.com/Leo-Poon-Lab/mutations-under-sarscov2-vaccination/blob/main/metadata/df_samples_anonymised.csv). SARS-CoV-2 reference genome (Wuhan-Hu-1, GenBank: MN908947.3) is available on GenBank. The SARS-CoV-2 CD8^+^ and CD4^+^ T cell epitope data were retrieved from the dashboard reported by us^48^ (https://www.mckayspcb.com/SARS2TcellEpitopes/; accessed on 15 November 2022) and the Immune Epitope Database (IEDB)^49^ (https://www.iedb.org; accessed on 15 November 2022). Source data are provided with this paper.

## Code availability

Detailed scripts for the above analysis are available from https://github.com/Leo-Poon-Lab/mutations-under-sarscov2-vaccination (10.5281/zenodo.7669077).
