# Supplementary material for: Within-host genetic diversity of SARS-CoV-2 lineages in unvaccinated and vaccinated individuals
Source: Nat Commun. 2023 Mar 31;14:1793. doi: 10.1038/s41467-023-37468-y (PMC10063955; doi:10.1038/s41467-023-37468-y)

Supplementary Tables

Supplementary Table 1. Incidence of iSNVs in different regions.

Gene	Mean iSNVs per Kb
ORF7a	0.209363186
ORF7b	0.219520432
N	0.273285563
ORF3a	0.308310091
ORF10	0.31823968
ORF8	0.32245861
Full genome	0.345495373
ORF1ab	0.346999125
ORF6	0.347405289
M	0.397463871
S	0.432841431
E	0.996240745

Supplementary Table 2. Distribution of high-frequent iSNVs in different viral lineage and vaccination groups.

Lineage	Vaccine	Gene	Mutation (amino acid)	Proportion
20A (B.1.36.*)	Unvaccinated	E	V75A	0.566225166
20B (B.1.1.63)	Unvaccinated	E	V75A	0.357615894
20I (Alpha)	Unvaccinated	E	V75A	0.049668874
21M (Delta, B.1.617.2.*)	BioNTech	E	V75A	0.009933775
21M (Delta, B.1.617.2.*)	Unvaccinated	E	V75A	0.006622517
21M (Omicron, BA.2.*)	Sinovac	E	V75A	0.006622517
21M (Delta, B.1.617.2.*)	Sinovac	E	V75A	0.003311258
20A (B.1.36.*)	Unvaccinated	S	K811R	0.515418502
20B (B.1.1.63)	Unvaccinated	S	K811R	0.31277533
20I (Alpha)	Unvaccinated	S	K811R	0.092511013
21M (Delta, B.1.617.2.*)	Unvaccinated	S	K811R	0.04845815
21M (Delta, B.1.617.2.*)	BioNTech	S	K811R	0.026431718
21M (Delta, B.1.617.2.*)	Sinovac	S	K811R	0.004405286
21M (Omicron, BA.2.*)	BioNTech	ORF1ab	Q1522Q	0.494949495
21M (Omicron, BA.2.*)	Sinovac	ORF1ab	Q1522Q	0.227272727
21M (Omicron, BA.2.*)	Unvaccinated	ORF1ab	Q1522Q	0.126262626
21M (Delta, B.1.617.2.*)	BioNTech	ORF1ab	Q1522Q	0.101010101
21M (Delta, B.1.617.2.*)	Unvaccinated	ORF1ab	Q1522Q	0.035353535
21M (Delta, B.1.617.2.*)	Sinovac	ORF1ab	Q1522Q	0.015151515
21M (Omicron, BA.2.*)	BioNTech	S	Q493Q	0.632183908
21M (Omicron, BA.2.*)	Sinovac	S	Q493Q	0.235632184
21M (Omicron, BA.2.*)	Unvaccinated	S	Q493Q	0.132183908
21M (Omicron, BA.2.*)	BioNTech	ORF1ab	A5447V	0.403508772
21M (Omicron, BA.2.*)	Sinovac	ORF1ab	A5447V	0.233918129
21M (Omicron, BA.2.*)	Unvaccinated	ORF1ab	A5447V	0.16374269
21M (Delta, B.1.617.2.*)	BioNTech	ORF1ab	A5447V	0.105263158
21M (Delta, B.1.617.2.*)	Unvaccinated	ORF1ab	A5447V	0.070175439
21M (Delta, B.1.617.2.*)	Sinovac	ORF1ab	A5447V	0.023391813

Supplementary Table 3. Differences in iSNVs between groups at full-genome level. Only pairs with significant difference (Benjamini-Hochberg (BH) adjusted P-values for multiple comparisons p<0.05, two-sided Wilcoxon Rank Sum test) are shown.

Variable 1	Variable 2	Median of variable 1	Median of variable 2	P value
**Number of iSNVs per Kb (adjusted)**				
Comirnaty_Doses2_21J (Delta, B.1.617.2.*)	Unvaccinated_21J (Delta, B.1.617.2.*)	0.1959	0.0192	0.0070
Comirnaty_Doses2_21M (Omicron, BA.2.*)	Comirnaty_Doses3_21M (Omicron, BA.2.*)	0.0232	-0.1630	0.0000
CoronaVac_Doses2_21M (Omicron, BA.2.*)	Comirnaty_Doses3_21M (Omicron, BA.2.*)	0.0292	-0.1630	0.0016
Comirnaty_Doses2_21J (Delta, B.1.617.2.*)	Comirnaty_Doses2_21M (Omicron, BA.2.*)	0.1959	0.0232	0.0016
Unvaccinated_20A (B.1.36.*)	Unvaccinated_20I (Alpha)	-0.1341	-0.0597	0.0064
Unvaccinated_20A (B.1.36.*)	Unvaccinated_21J (Delta, B.1.617.2.*)	-0.1341	0.0192	0.0001
Unvaccinated_20B (B.1.1.63)	Unvaccinated_20I (Alpha)	-0.1357	-0.0597	0.0006
Unvaccinated_20B (B.1.1.63)	Unvaccinated_21J (Delta, B.1.617.2.*)	-0.1357	0.0192	0.0000
Unvaccinated_20B (B.1.1.63)	Unvaccinated_21M (Omicron, BA.2.*)	-0.1357	-0.0598	0.0185
**Minor allele frequency (adjusted)**				
NA				
**Nucleotide diversity (π) (adjusted)**				
Comirnaty_Doses2_21M (Omicron, BA.2.*)	Comirnaty_Doses3_21M (Omicron, BA.2.*)	5.20E-06	-3.73E-05	0.0000
CoronaVac_Doses2_21M (Omicron, BA.2.*)	Comirnaty_Doses3_21M (Omicron, BA.2.*)	7.50E-06	-3.73E-05	0.0004
CoronaVac_Doses3_21M (Omicron, BA.2.*)	Comirnaty_Doses3_21M (Omicron, BA.2.*)	-8.82E-06	-3.73E-05	0.0189
Unvaccinated_21J (Delta, B.1.617.2.*)	Comirnaty_Doses2_21J (Delta, B.1.617.2.*)	-2.02E-06	7.29E-05	0.0137
Unvaccinated_21M (Omicron, BA.2.*)	Comirnaty_Doses3_21M (Omicron, BA.2.*)	-3.02E-06	-3.73E-05	0.0070
Comirnaty_Doses2_21J (Delta, B.1.617.2.*)	Comirnaty_Doses2_21M (Omicron, BA.2.*)	7.29E-05	5.20E-06	0.0011
Unvaccinated_20A (B.1.36.*)	Unvaccinated_20I (Alpha)	-3.16E-05	-5.53E-06	0.0048
Unvaccinated_20A (B.1.36.*)	Unvaccinated_21J (Delta, B.1.617.2.*)	-3.16E-05	-2.02E-06	0.0024
Unvaccinated_20B (B.1.1.63)	Unvaccinated_20I (Alpha)	-3.28E-05	-5.53E-06	0.0002
Unvaccinated_20B (B.1.1.63)	Unvaccinated_21J (Delta, B.1.617.2.*)	-3.28E-05	-2.02E-06	0.0001

Supplementary Table 4. Synonymous and nonsynonymous nucleotide diversity on full genome and spike gene of different groups. P value was evaluated using two-sided Z-tests of the null hypothesis that $\pi_N - \pi_S = 0$ (10,000 bootstrap replicates, codon unit).

Gene	Group	π_N ($\pm SD$) (10^{-5})	π_S ($\pm SD$) (10^{-5})	$\pi_N - \pi_S$ (10^{-5})	P value
Full genome	Combined	0.25 (0.1~0.4)	0.4 (0.08~0.72)	-0.15	0.6775
Full genome	Comirnaty (Doses=2)<br>21J (Delta, B.1.617.2.*)	3.08 (2.62~3.54)	4.97 (3.96~5.98)	-1.89	0.0925
Full genome	Comirnaty (Doses=2)<br>21M (Omicron, BA.2.*)	1.34 (1~1.68)	3.31 (2.12~4.5)	-1.97	0.1074
Full genome	Comirnaty (Doses=3)<br>21M (Omicron, BA.2.*)	-1.42 (-1.69~-1.15)	-1.43 (-2.04~-0.82)	0.01	0.985
Full genome	CoronaVac (Doses=2)<br>21J (Delta, B.1.617.2.*)	3.61 (3.04~4.18)	6.03 (4.58~7.48)	-2.42	0.1231
Full genome	CoronaVac (Doses=2)<br>21M (Omicron, BA.2.*)	0.82 (0.49~1.15)	3.24 (2.22~4.26)	-2.42	0.0228
Full genome	CoronaVac (Doses=3)<br>21M (Omicron, BA.2.*)	0.15 (-0.15~0.45)	0.99 (0.19~1.79)	-0.85	0.3161
Full genome	Unvaccinated<br>20A (B.1.36.*)	0.09 (-0.11~0.29)	-0.02 (-0.5~0.46)	0.11	0.8227
Full genome	Unvaccinated<br>20B (B.1.1.63)	-0.46 (-0.59~-0.33)	-1.41 (-1.59~-1.23)	0.95	0
Full genome	Unvaccinated<br>20I (Alpha)	1.75 (1.37~2.13)	1.93 (1.35~2.51)	-0.18	0.7963
Full genome	Unvaccinated<br>21J (Delta, B.1.617.2.*)	2.91 (2.5~3.32)	3.84 (3.06~4.62)	-0.93	0.3005
Full genome	Unvaccinated<br>21M (Omicron, BA.2.*)	1.48 (1.09~1.87)	4.02 (2.93~5.11)	-2.54	0.0276
S	Combined	1.07 (0.62~1.52)	1.77 (0.43~3.11)	-0.7	0.6143
S	Comirnaty (Doses=2)<br>21J (Delta, B.1.617.2.*)	2.71 (1.84~3.58)	5.06 (2.87~7.25)	-2.35	0.3203
S	Comirnaty (Doses=2)<br>21M (Omicron, BA.2.*)	1.77 (0.8~2.74)	2.75 (1.25~4.25)	-0.98	0.5808
S	Comirnaty (Doses=3)<br>21M (Omicron, BA.2.*)	0.06 (-1.63~1.75)	-1.07 (-2.64~0.5)	1.13	0.6272
S	CoronaVac (Doses=2)<br>21J (Delta, B.1.617.2.*)	3.24 (1.76~4.72)	10.16 (4.66~15.66)	-6.92	0.2175
S	CoronaVac (Doses=2)<br>21M (Omicron, BA.2.*)	0.06 (-0.83~0.95)	3.38 (1.34~5.42)	-3.32	0.1367
S	CoronaVac (Doses=3)<br>21M (Omicron, BA.2.*)	0.97 (-0.29~2.23)	3.48 (0.9~6.06)	-2.51	0.38
S	Unvaccinated<br>20A (B.1.36.*)	1.21 (0.69~1.73)	3.06 (0.05~6.07)	-1.84	0.5505
S	Unvaccinated<br>20B (B.1.1.63)	0.2 (-0.18~0.58)	-1.3 (-1.72~-0.88)	1.51	0.0067
S	Unvaccinated<br>20I (Alpha)	3.73 (2.02~5.44)	2.1 (0.81~3.39)	1.63	0.442
S	Unvaccinated<br>21J (Delta, B.1.617.2.*)	3.74 (2.66~4.82)	3.56 (1.87~5.25)	0.18	0.9297
S	Unvaccinated<br>21M (Omicron, BA.2.*)	1.91 (0.91~2.91)	4.81 (2.24~7.38)	-2.91	0.2863

Supplementary Table 5. Number of total analysed samples, stratified by virus lineages and vaccination status.

	Comirnaty	CoronaVac	Unvaccinated
**20A (B.1.36.*)**	0	0	1116
**20B (B.1.1.63)**	0	0	863
**20I (Alpha)**	0	0	77
**21J (Delta, B.1.617.2.*)**	66	17	63
**21M (Omicron, BA.2.*)**	355	172	91

Supplementary Table 6. Optimal MAF thresholds for iSNVs detection for depth cutoff of 100 reads. The optimal thresholds were determined by Youden's J statistic (Youden, 1950). The optimal cut-off is the threshold that maximizes the distance to the identity (diagonal) line.

sample	threshold	specificity	sensitivity	AUC
e-1	0.02	0.9375	0.7	0.865625
e-2	0.02	0.857142857	0.6	0.802380952
e-3	0.015	1	0.65	0.814285714
e-5	0.025	0.980769231	0.6	0.855769231
e-6-1 (Ct:24.78)	0.025	0.976190476	0.65	0.715972222
e-6-2 (Ct:25.36)	0.025	0.993127148	0.55	0.661082474
e-7-1 (Ct:28.36)	0.02	0.976190476	0.2	0.198809524
e-7-2 (Ct:28.59)	0.03	0.989361702	0.1	0.108510638

Supplementary Table 7. Number of analysed samples, stratified by virus lineages, vaccination status and specimen types.

Vaccine	lineages	Specimen type	Number
Comirnaty (Doses=2)	21J (Delta, B.1.617.2.*)	Deep throat saliva	4
Comirnaty (Doses=2)	21J (Delta, B.1.617.2.*)	NPS + Throat swab	15
Comirnaty (Doses=2)	21J (Delta, B.1.617.2.*)	Throat and nasal swab	39
Comirnaty (Doses=2)	21J (Delta, B.1.617.2.*)	Throat saliva	8
Comirnaty (Doses=2)	21M (Omicron, BA.2.*)	Deep throat saliva	4
Comirnaty (Doses=2)	21M (Omicron, BA.2.*)	Nasal swab	82
Comirnaty (Doses=2)	21M (Omicron, BA.2.*)	Nasopharyngeal aspirate	1
Comirnaty (Doses=2)	21M (Omicron, BA.2.*)	Nasopharyngeal swab	1
Comirnaty (Doses=2)	21M (Omicron, BA.2.*)	NPA + Throat swab	1
Comirnaty (Doses=2)	21M (Omicron, BA.2.*)	NPS + Throat swab	6
Comirnaty (Doses=2)	21M (Omicron, BA.2.*)	Throat and nasal swab	80
Comirnaty (Doses=2)	21M (Omicron, BA.2.*)	Throat saliva	18
Comirnaty (Doses=3)	21M (Omicron, BA.2.*)	Deep throat saliva	1
Comirnaty (Doses=3)	21M (Omicron, BA.2.*)	Nasal swab	78
Comirnaty (Doses=3)	21M (Omicron, BA.2.*)	NPS + Throat swab	1
Comirnaty (Doses=3)	21M (Omicron, BA.2.*)	Throat and nasal swab	15
Comirnaty (Doses=3)	21M (Omicron, BA.2.*)	Throat saliva	7
CoronaVac (Doses=2)	21J (Delta, B.1.617.2.*)	Deep throat saliva	1
CoronaVac (Doses=2)	21J (Delta, B.1.617.2.*)	NPS + Throat swab	4
CoronaVac (Doses=2)	21J (Delta, B.1.617.2.*)	Throat and nasal swab	8
CoronaVac (Doses=2)	21J (Delta, B.1.617.2.*)	Throat saliva	4

CoronaVac (Doses=2)	21M (Omicron, BA.2.*)	Deep throat saliva	6
CoronaVac (Doses=2)	21M (Omicron, BA.2.*)	Nasal swab	29
CoronaVac (Doses=2)	21M (Omicron, BA.2.*)	Nasopharyngeal swab	1
CoronaVac (Doses=2)	21M (Omicron, BA.2.*)	Nephrostomy urine	1
CoronaVac (Doses=2)	21M (Omicron, BA.2.*)	NPS + Throat swab	6
CoronaVac (Doses=2)	21M (Omicron, BA.2.*)	Sputum	1
CoronaVac (Doses=2)	21M (Omicron, BA.2.*)	Throat and nasal swab	40
CoronaVac (Doses=2)	21M (Omicron, BA.2.*)	Throat saliva	10
CoronaVac (Doses=3)	21M (Omicron, BA.2.*)	Nasal swab	23
CoronaVac (Doses=3)	21M (Omicron, BA.2.*)	Nasopharyngeal swab	1
CoronaVac (Doses=3)	21M (Omicron, BA.2.*)	Throat and nasal swab	18
CoronaVac (Doses=3)	21M (Omicron, BA.2.*)	Throat saliva	2
Unvaccinated	20A (B.1.36.*)	Deep throat saliva	61
Unvaccinated	20A (B.1.36.*)	Nasopharyngeal aspirate	2
Unvaccinated	20A (B.1.36.*)	Nasopharyngeal swab	93
Unvaccinated	20A (B.1.36.*)	NPA + Throat swab	41
Unvaccinated	20A (B.1.36.*)	NPS + Throat swab	157
Unvaccinated	20A (B.1.36.*)	Sputum	7
Unvaccinated	20A (B.1.36.*)	Throat and nasal swab	271
Unvaccinated	20A (B.1.36.*)	Throat saliva	474
Unvaccinated	20A (B.1.36.*)	Throat swab	4
Unvaccinated	20B (B.1.1.63)	Nasopharyngeal aspirate	1
Unvaccinated	20B (B.1.1.63)	Nasopharyngeal swab	34
Unvaccinated	20B (B.1.1.63)	NPA + Throat swab	42
Unvaccinated	20B (B.1.1.63)	NPS + Throat swab	170
Unvaccinated	20B (B.1.1.63)	Sputum	13
Unvaccinated	20B (B.1.1.63)	Throat and nasal swab	18
Unvaccinated	20B (B.1.1.63)	Throat saliva	574
Unvaccinated	20B (B.1.1.63)	Throat swab	1
Unvaccinated	20B (B.1.1.63)	Tracheal aspirate	2
Unvaccinated	20I (Alpha)	Nasopharyngeal aspirate	1
Unvaccinated	20I (Alpha)	Nasopharyngeal swab	2
Unvaccinated	20I (Alpha)	NPA + Throat swab	4
Unvaccinated	20I (Alpha)	NPS + Throat swab	4
Unvaccinated	20I (Alpha)	Throat and nasal swab	47
Unvaccinated	20I (Alpha)	Throat saliva	15
Unvaccinated	21J (Delta, B.1.617.2.*)	Deep throat saliva	8
Unvaccinated	21J (Delta, B.1.617.2.*)	Nasopharyngeal swab	2
Unvaccinated	21J (Delta, B.1.617.2.*)	NPA + Throat swab	1
Unvaccinated	21J (Delta, B.1.617.2.*)	NPS + Throat swab	16
Unvaccinated	21J (Delta, B.1.617.2.*)	Throat and nasal swab	26
Unvaccinated	21J (Delta, B.1.617.2.*)	Throat saliva	9
Unvaccinated	21M (Omicron, BA.2.*)	Deep throat saliva	5
Unvaccinated	21M (Omicron, BA.2.*)	Nasal swab	20
Unvaccinated	21M (Omicron, BA.2.*)	Nasopharyngeal swab	2
Unvaccinated	21M (Omicron, BA.2.*)	NPA + Throat swab	1

Unvaccinated	21M (Omicron, BA.2.*)	NPS + Throat swab	10
Unvaccinated	21M (Omicron, BA.2.*)	Throat and nasal swab	26
Unvaccinated	21M (Omicron, BA.2.*)	Throat saliva	8

Supplementary Figures

Supplementary Figure 1. The sequencing depth in sliding window of 200bp of the samples included in this study. Each grey line is representative of one individual sample, and the red line shows the average of all samples. The dashed line showed depth of 100 reads. Source data are provided as a Source Data file.

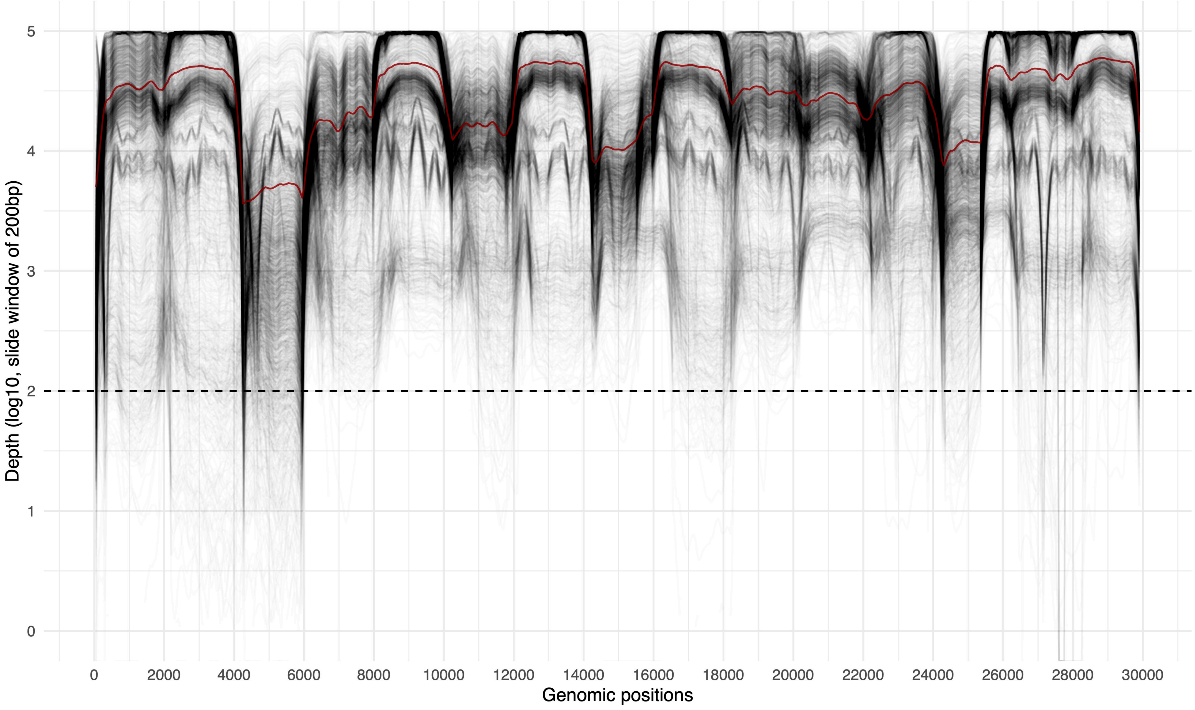

Supplementary Figure 2. Correlation of Ct value between different factors. The regression lines are showed in blue. **(A)** Correlation between Ct value and number of iSNVs per Kb; **(B)** Correlation between Ct value and adjusted number of iSNVs per Kb; **(C)** Correlation between Ct value and nucleotide diversity; **(D)** Correlation between Ct value and adjusted nucleotide diversity; **(E)** Correlation between detection lag (time post symptom onset in days) and number of iSNVs per Kb; **(F)** Correlation between detection lag and adjusted number of iSNVs per Kb. Statistical test used is two-sided Pearson correlation test with no adjustment for multiple comparisons. The error bands in grey show the 95% confidence interval for linear regression. Source data are provided as a Source Data file.

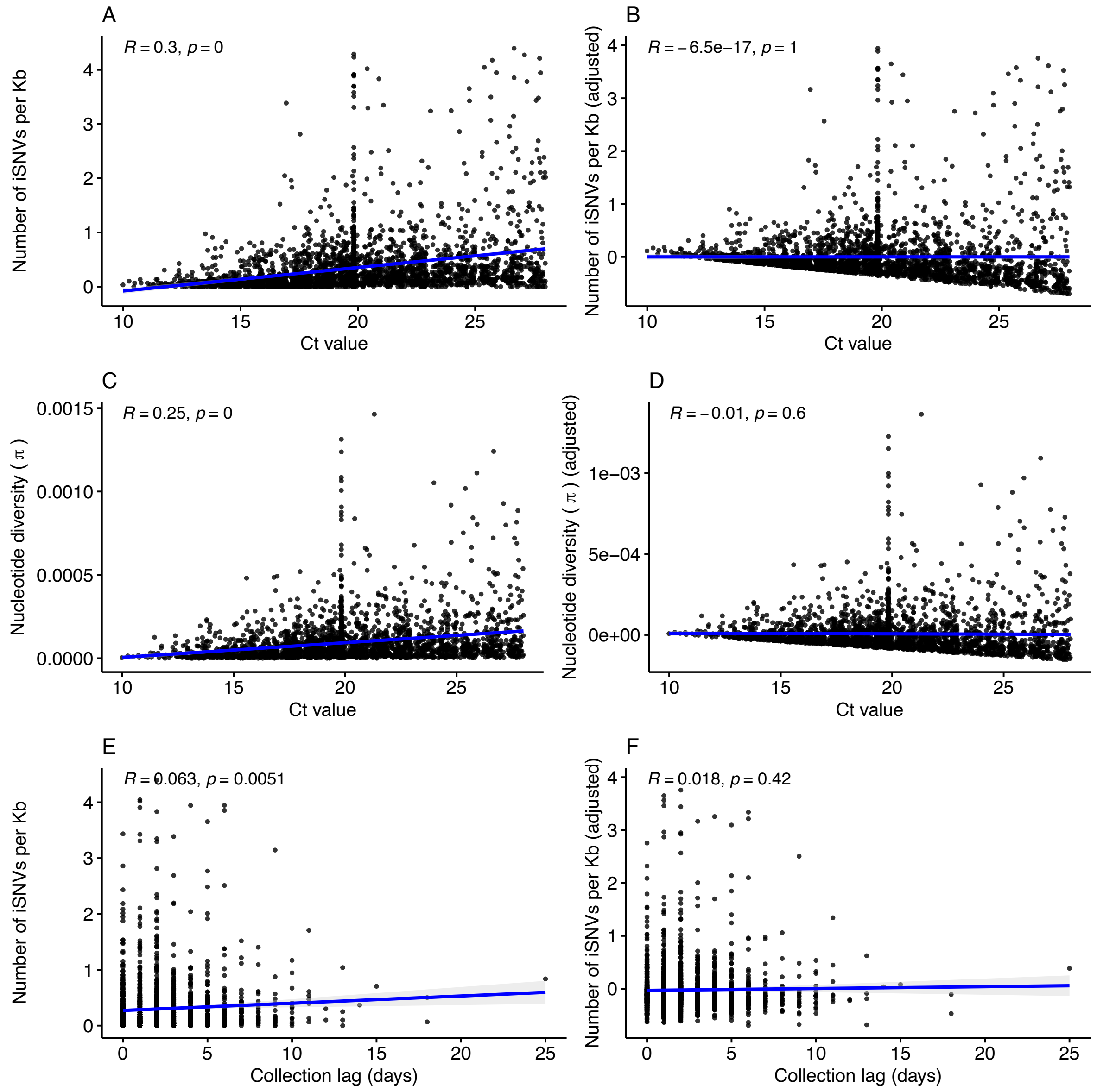

Supplementary Figure 3. Within-host mutations profiles among different groups. Different vaccination statuses were compared, the data used here are the same as the data used in the Figure 2A-2C and Figure 3A-3C of the main text. Boxplots indicate median and inter-quartile ranges (IQR), and whiskers represent value ranges up to 1.5 * IQR. Pairwise comparisons within groups were tested by two-sided two-sample Wilcoxon tests, the pairs with Benjamini-Hochberg (BH) adjusted P value ≤ 0.01 and ≤ 0.05 were labelled with "**" and "*" respectively. The number of biologically independent samples in each group are shown in Supplementary Table 5. **(A)** Full-genome incidence of iSNVs (adjusted number of iSNVs per Kb) of different samples. **(B)** Full-genome abundance of iSNVs (minor allele frequencies) of different samples. **(C)** Full-genome adjusted nucleotide diversity (π) of different samples. Source data are provided as a Source Data file.

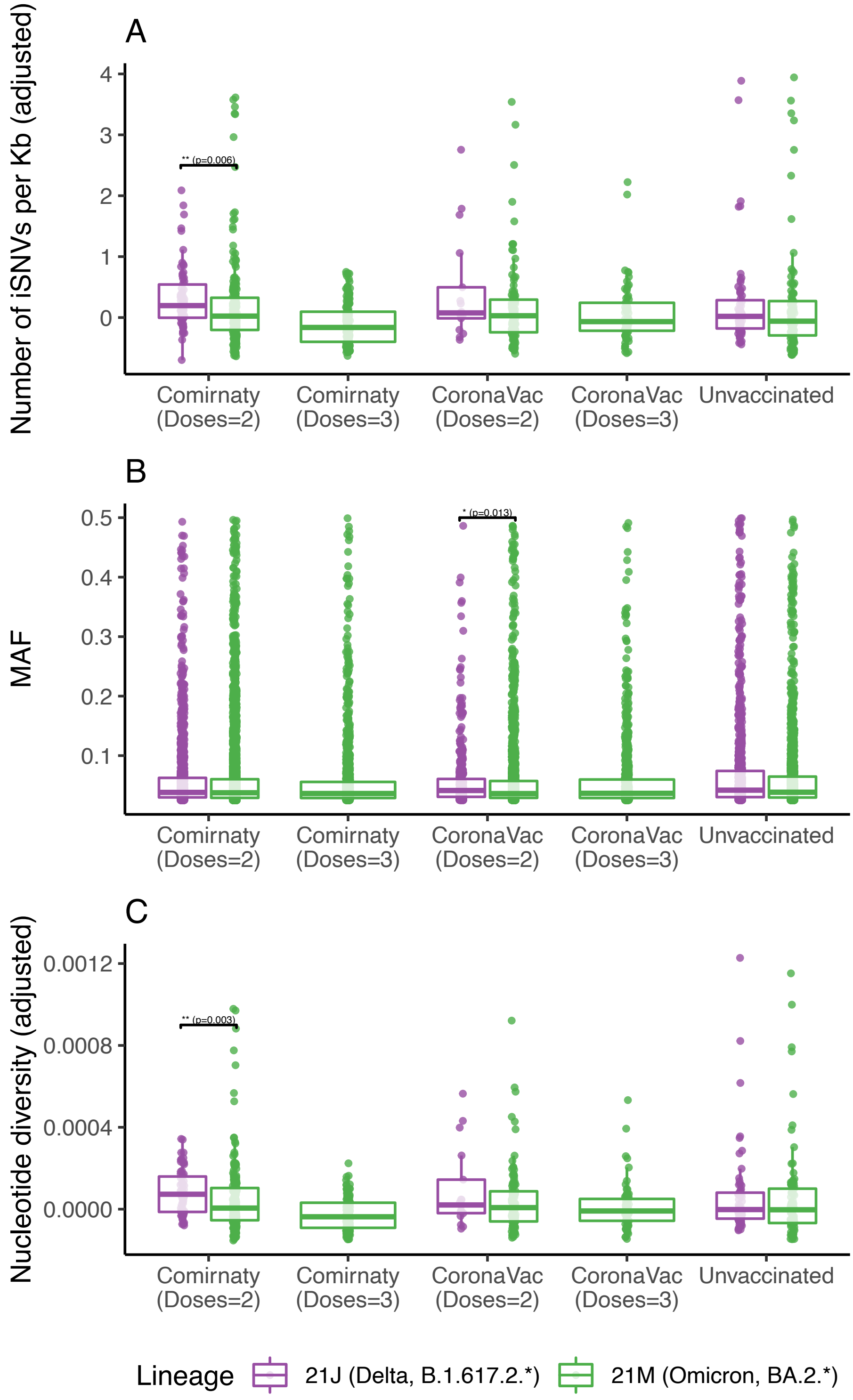

Supplementary Figure 4. Distribution of time post last dose in the vaccinated samples. Pairwise comparisons within groups were tested by two-sided two-sample Wilcoxon tests, the pairs with Benjamini-Hochberg (BH) adjusted P value ≤ 0.01 and ≤ 0.05 were labelled with "**" and "*" respectively. The number of biologically independent samples in each group are shown in Supplementary Table 5. For all box plots, the bold horizontal line inside the box shows the median, the upper and lower edges of the box indicate the first and the third quartiles, and whiskers extend to span a 1.5 interquartile range from the edges. Source data are provided as a Source Data file.

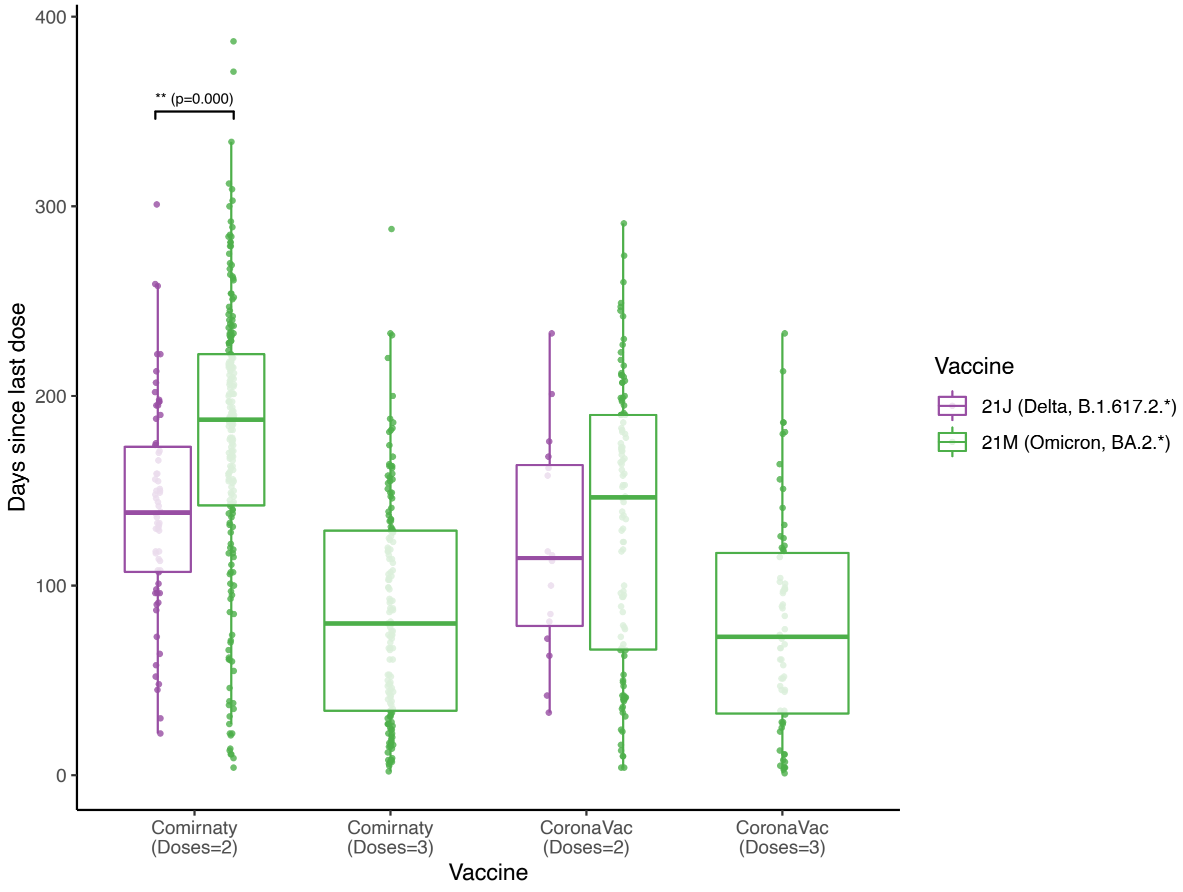

Supplementary Figure 5. Sliding window analysis of Synonymous/Non-synonymous nucleotide diversity in different genes. Sliding windows size of thirty codons and step size of one codon were used because this did not exceed the length of ORF10 (thirty-nine codons). Source data are provided as a Source Data file.

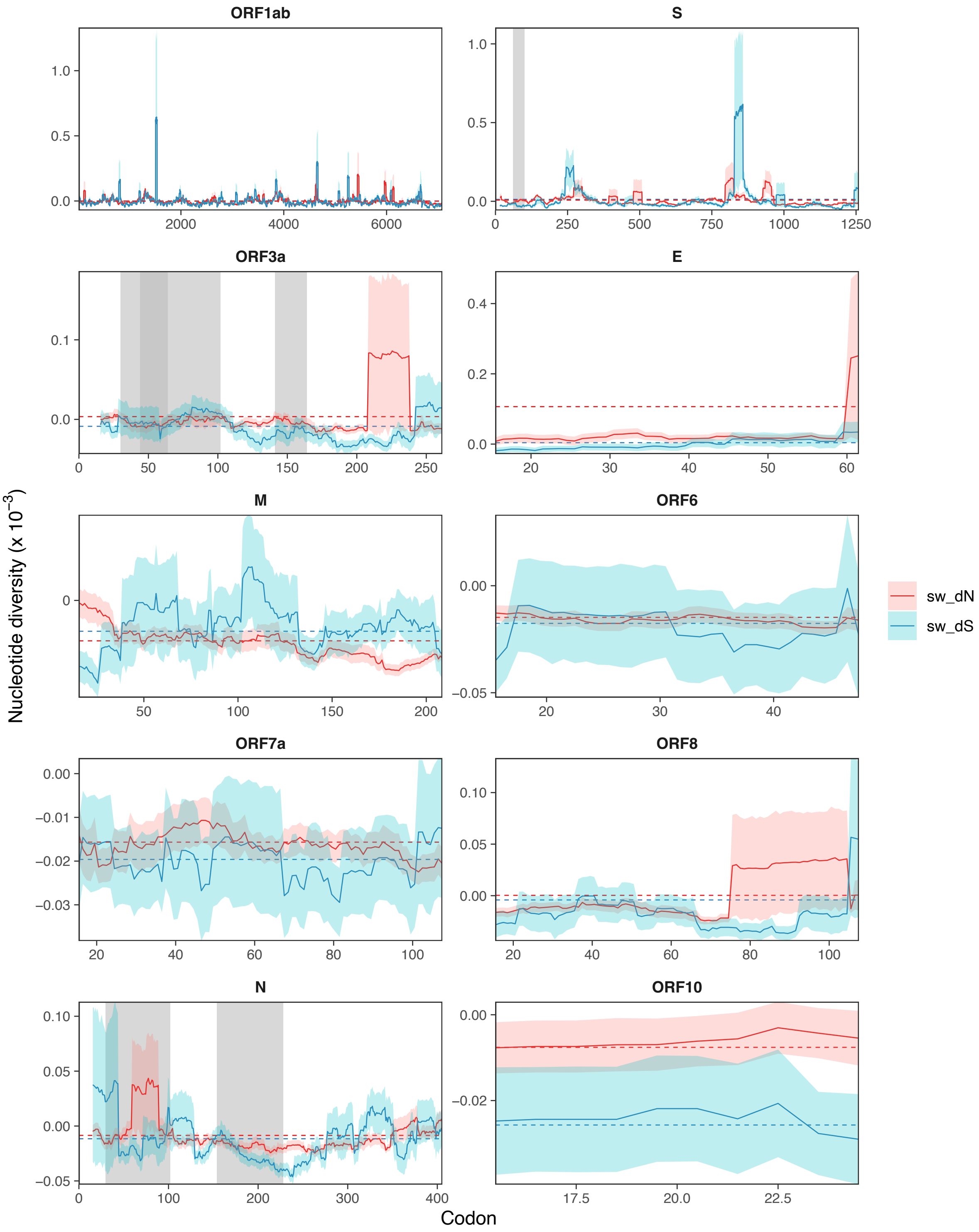

Supplementary Figure 6. Distribution of CD4+/CD8+ T cell epitope-HLA pairs included in this study. (A) Distribution in different genomic regions; **(B)** Distribution according to associated HLAs. Source data are provided as a Source Data file.

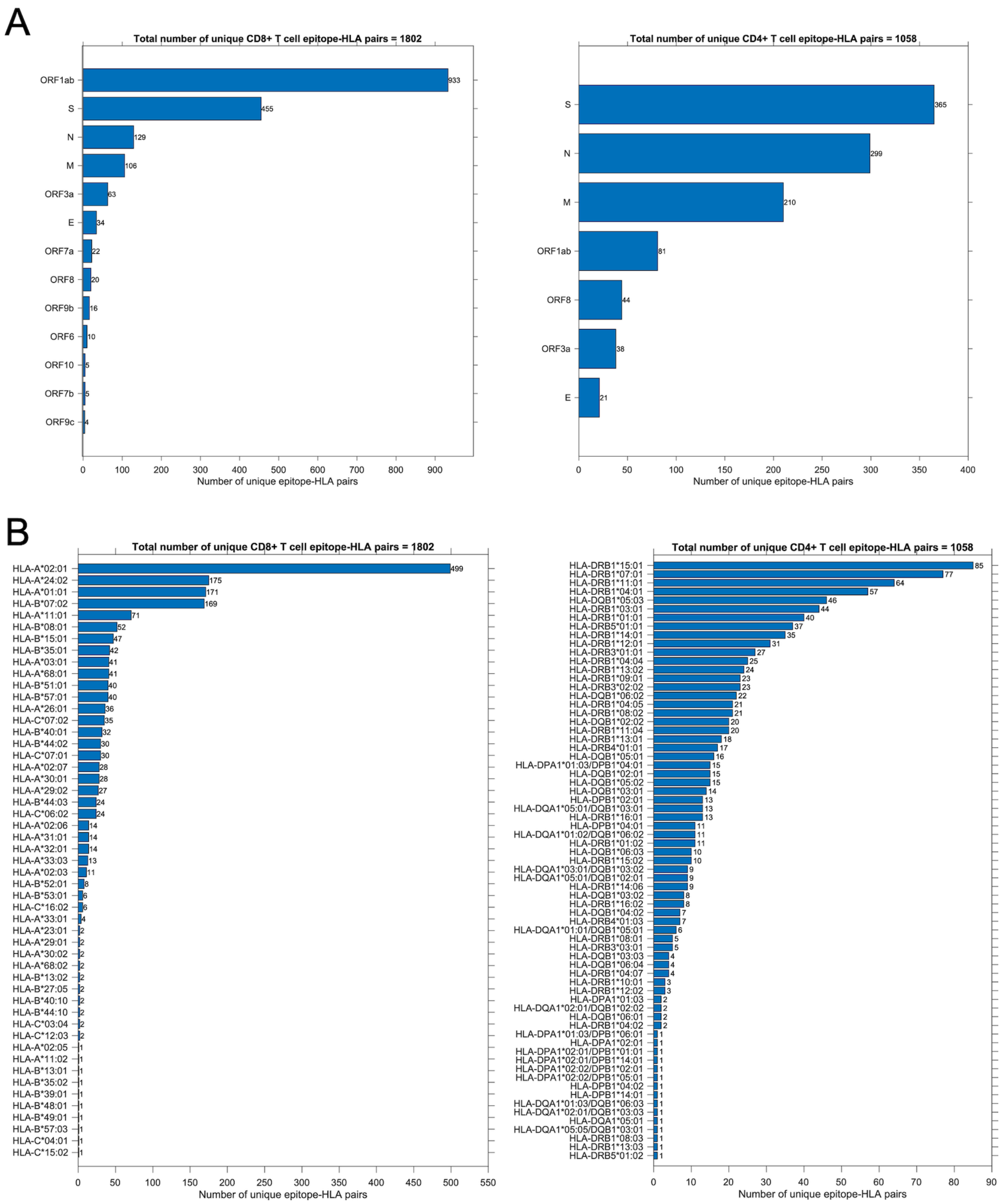

Supplementary Figure 7. Average number of overlapping epitopes per mutation in different groups. Source data are provided as a Source Data file.

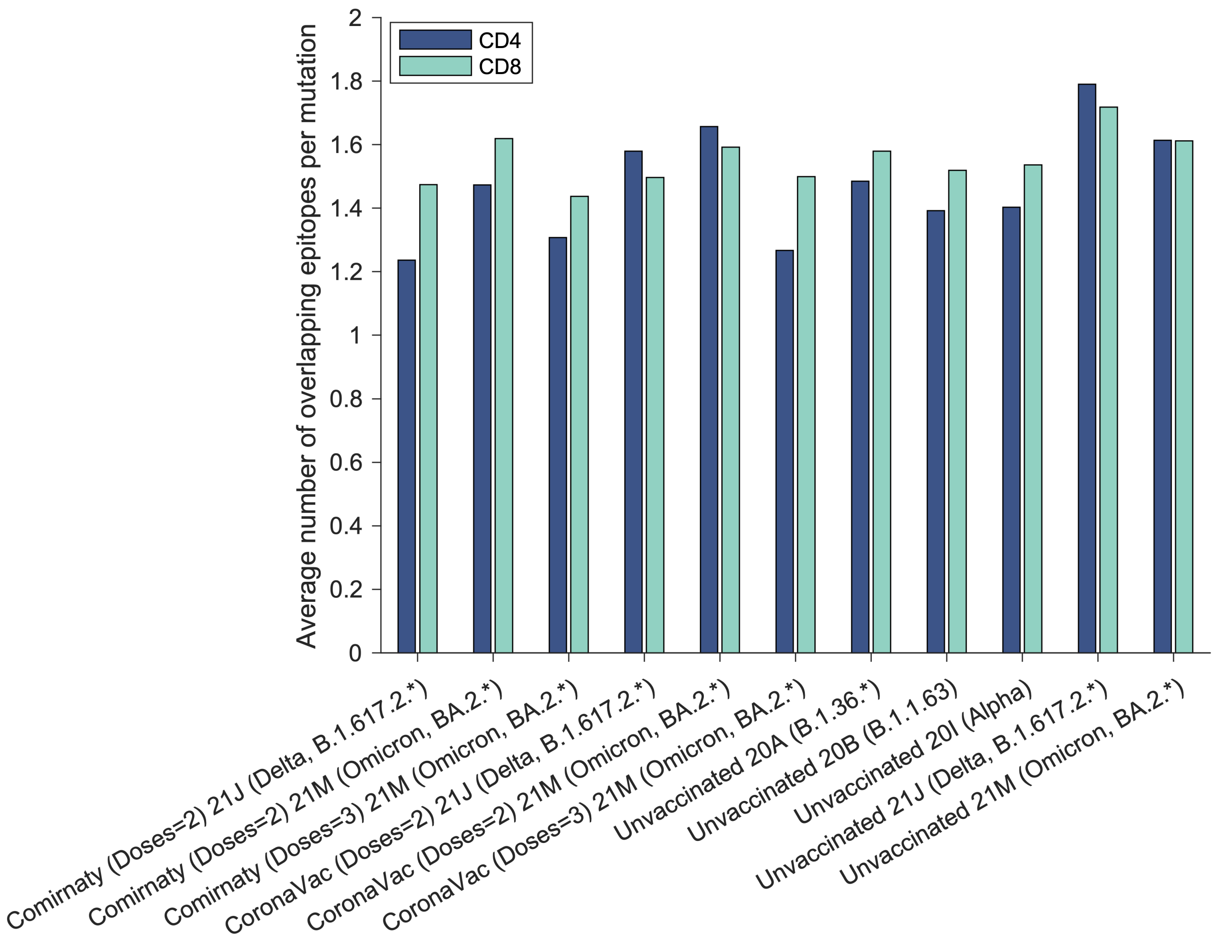

Supplementary Figure 8. Overlap of CD4+/CD8+ T cell epitopes per mutation in Spike between vaccinated and unvaccinated samples. (A) Analysis based on all available unique T cell epitopes; **(B)** Analysis based on epitope-HLA pairs specific to Hong Kong population. For all box plots, the bold horizontal line inside the box shows the median, the upper and lower edges of the box indicate the first and the third quartiles, and whiskers extend to span a 1.5 interquartile range from the edges. The sample size of data in each group are shown in Supplementary Figures 6 and 9. Source data are provided as a Source Data file.

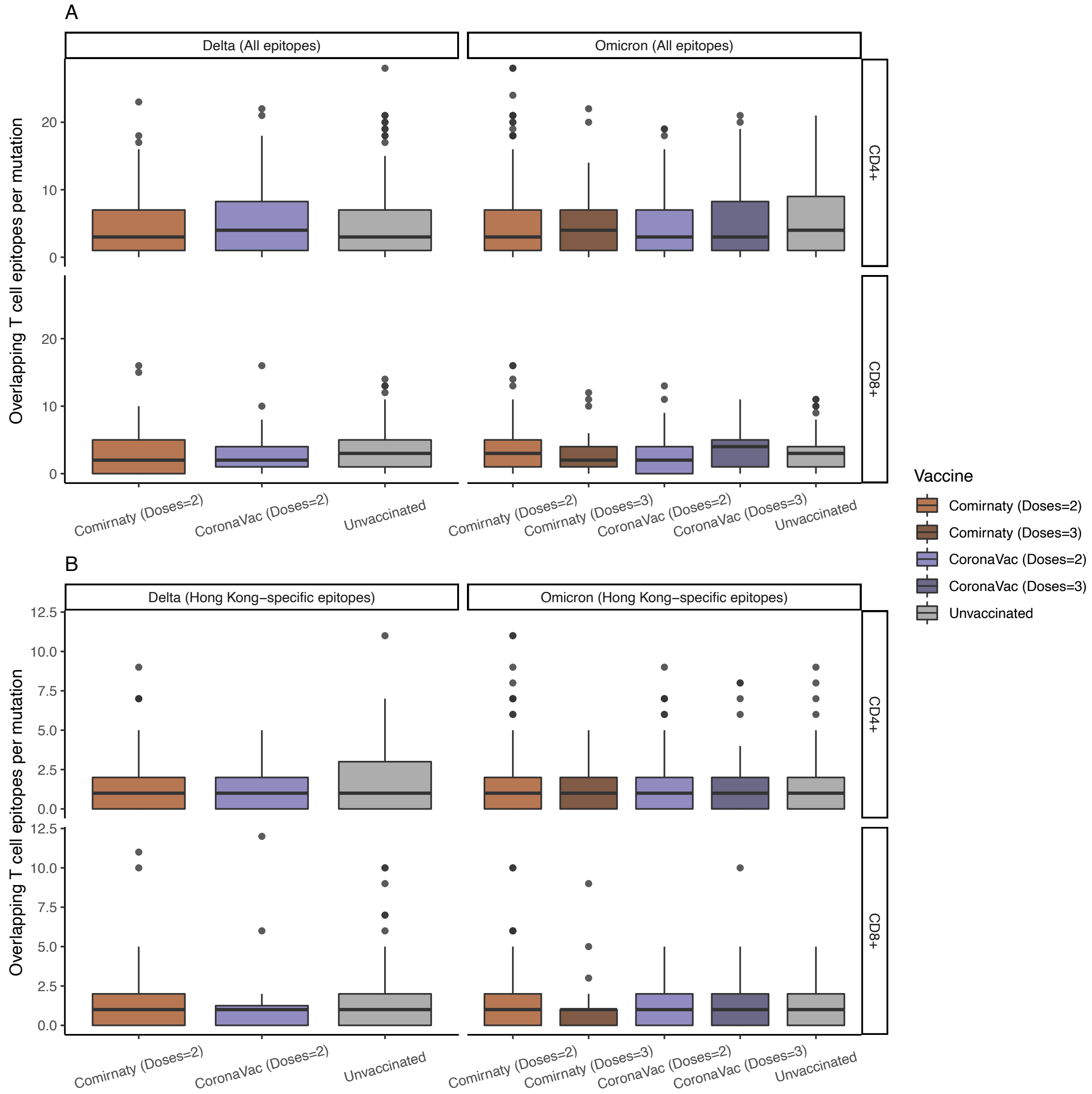

Supplementary Figure 9. Distribution of T cell epitope-HLA pairs specific to Hong Kong population. (**A**) Distribution of $CD8^{+}$ T cell epitopes; (**B**) Distribution of $CD4^{+}$ T cell epitopes. Source data are provided as a Source Data file.

A

No.	HLA allele	Coverage in HK population (%)#
1	HLA-A*11:01	49.12
2	HLA-A*24:02	28.4
3	HLA-B*40:01	28.01
4	HLA-A*02:07	24.46
5	HLA-A*33:03	18.98
6	HLA-A*02:03	14.98
7	HLA-B*13:01	14.95
8	HLA-A*02:01	12
9	HLA-A*02:06	9.17
10	HLA-B*51:01	8.39
11	HLA-A*11:02	7.83
12	HLA-B*15:01	4.34

#Source: IEDB

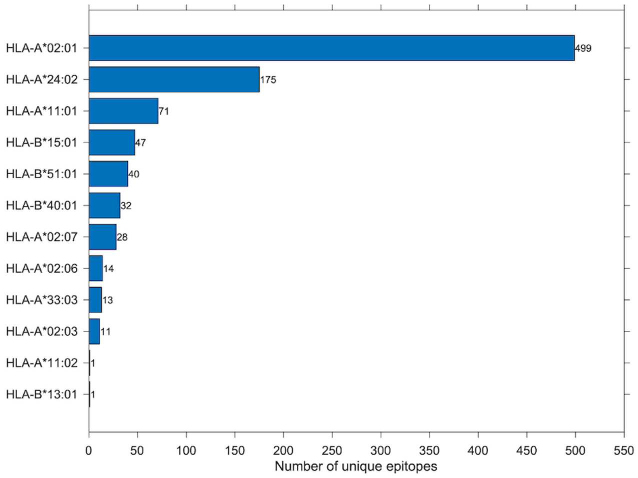

B

No.	HLA allele	Coverage in HK population (%)#
1	HLA-DQB1*03:01	23.55
2	HLA-DQB1*03:03	15.75
3	HLA-DRB1*09:01	15.37
4	HLA-DRB1*12:02	14.39
5	HLA-DQB1*05:02	11.76
6	HLA-DQB1*06:01	11.27
7	HLA-DRB1*15:01	9.65
8	HLA-DRB1*03:01	6.79
9	HLA-DQB1*02:01	6.69
10	HLA-DQB1*03:02	6.29
11	HLA-DRB1*04:05	6.21
12	HLA-DRB1*08:03	5.94
13	HLA-DRB1*11:01	5.3

#Source: AFND (Population coverage > 5%)

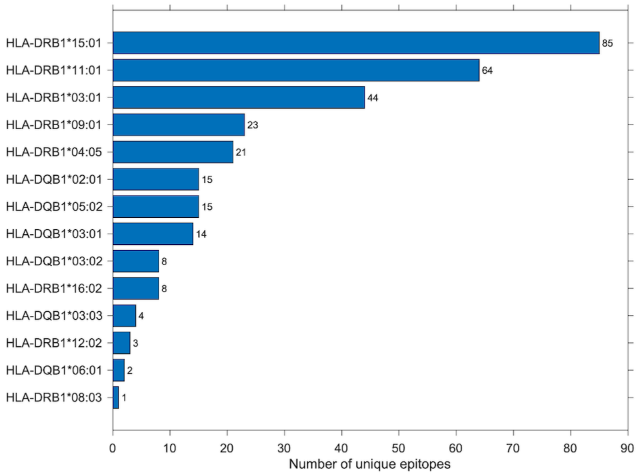

Supplementary Figure 10. Overlap of CD4+/CD8+ T cell epitopes per mutation at the individual HLA allele level. (**A**) P-values of pair-wise comparisons between vaccinated and unvaccinated samples for class I HLA alleles. (**B**) P-values of pair-wise comparisons between vaccinated and unvaccinated samples for class II HLA alleles. Pairwise comparisons within groups were performed by the two-sided two-sample Wilcoxon test. Analysis was performed for HLA alleles with at least 20 associated epitopes. Source data are provided as a Source Data file.

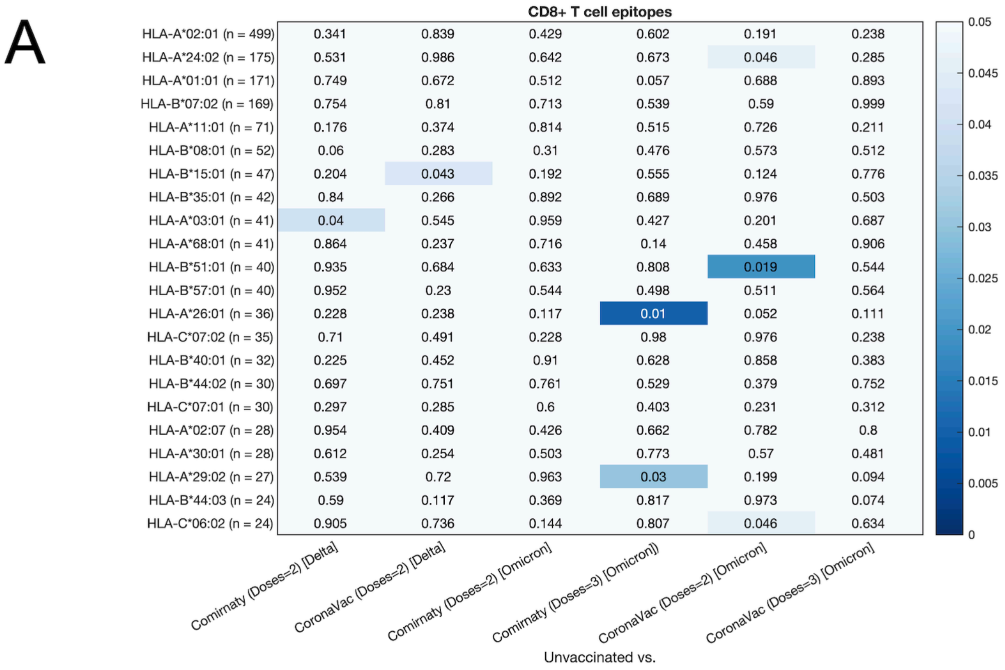

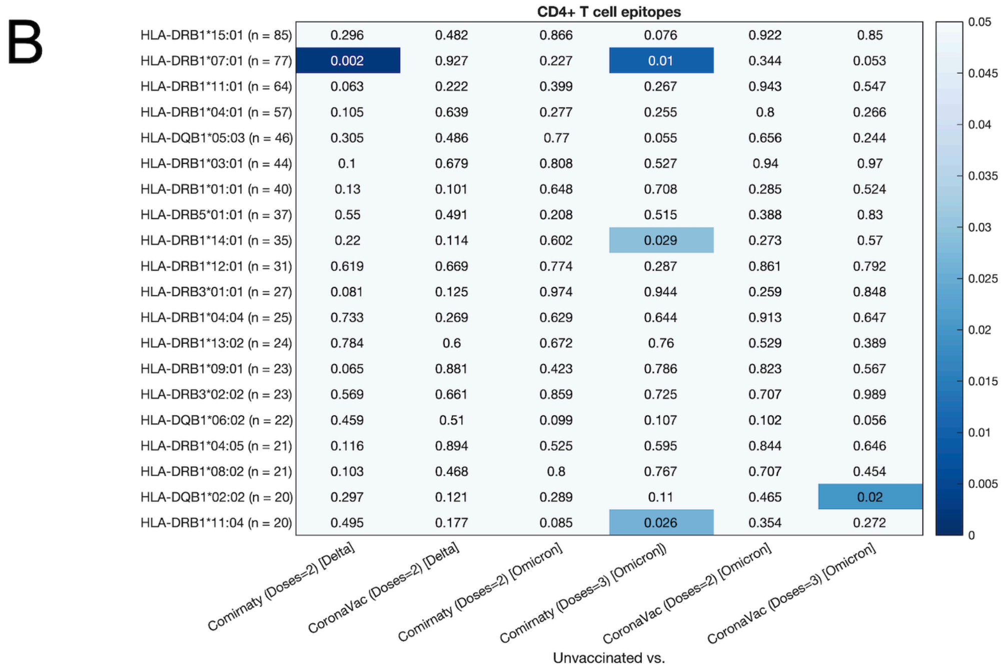

Supplementary Figure 11. Distribution of incidences of iSNVs (Ct value adjusted) between specimen types in different groups. Pairwise comparisons within groups were tested by two-sided two-sample Wilcoxon tests; the pairs with Benjamini-Hochberg (BH) adjusted P value ≤ 0.01 and ≤ 0.05 are labelled with "**" and "*" respectively. For all box plots, the bold horizontal line inside the box shows the median, the upper and lower edges of the box indicate the first and the third quartiles, and whiskers extend to span a 1.5 interquartile range from the edges. The number of biologically independent samples in each group are shown in Supplementary Table 7. Source data are provided as a Source Data file.

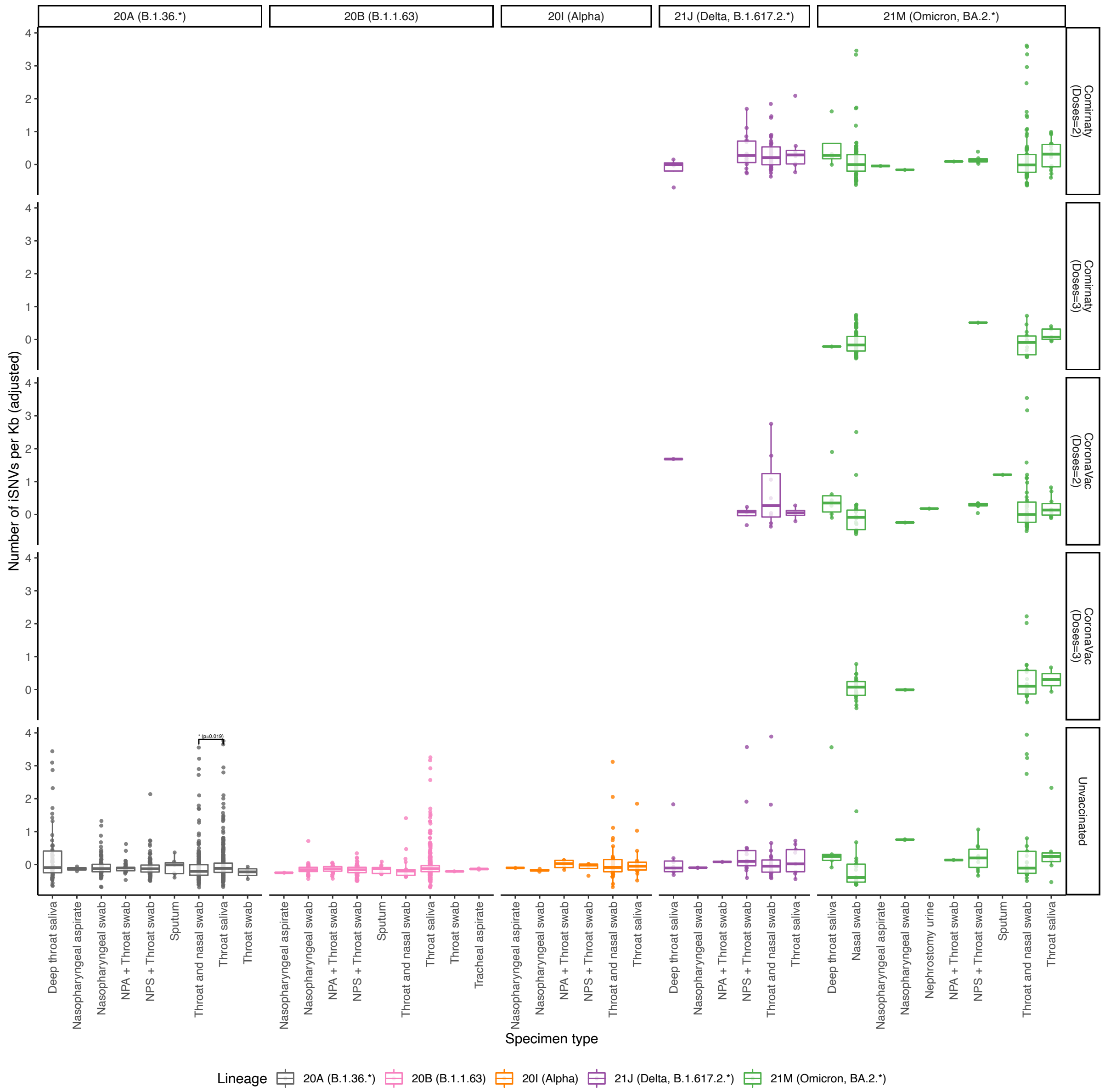

Supplementary Figure 12. Distribution of collection lag (days between symptom onset and sample collection) between different groups. (**A**) Distribution in unvaccinated samples; (**B**) Distribution in vaccinated samples. Pairwise comparisons within groups were tested by two-sided two-sample Wilcoxon tests; the pairs with Benjamini-Hochberg (BH) adjusted value ≤ 0.01 and ≤ 0.05 are labelled with "**" and "*" respectively. The number of biologically independent samples in each group are shown in Supplementary Table 5. For all box plots, the bold horizontal line inside the box shows the median, the upper and lower edges of the box indicate the first and the third quartiles, and whiskers extend to span a 1.5 interquartile range from the edges. Source data are provided as a Source Data file.

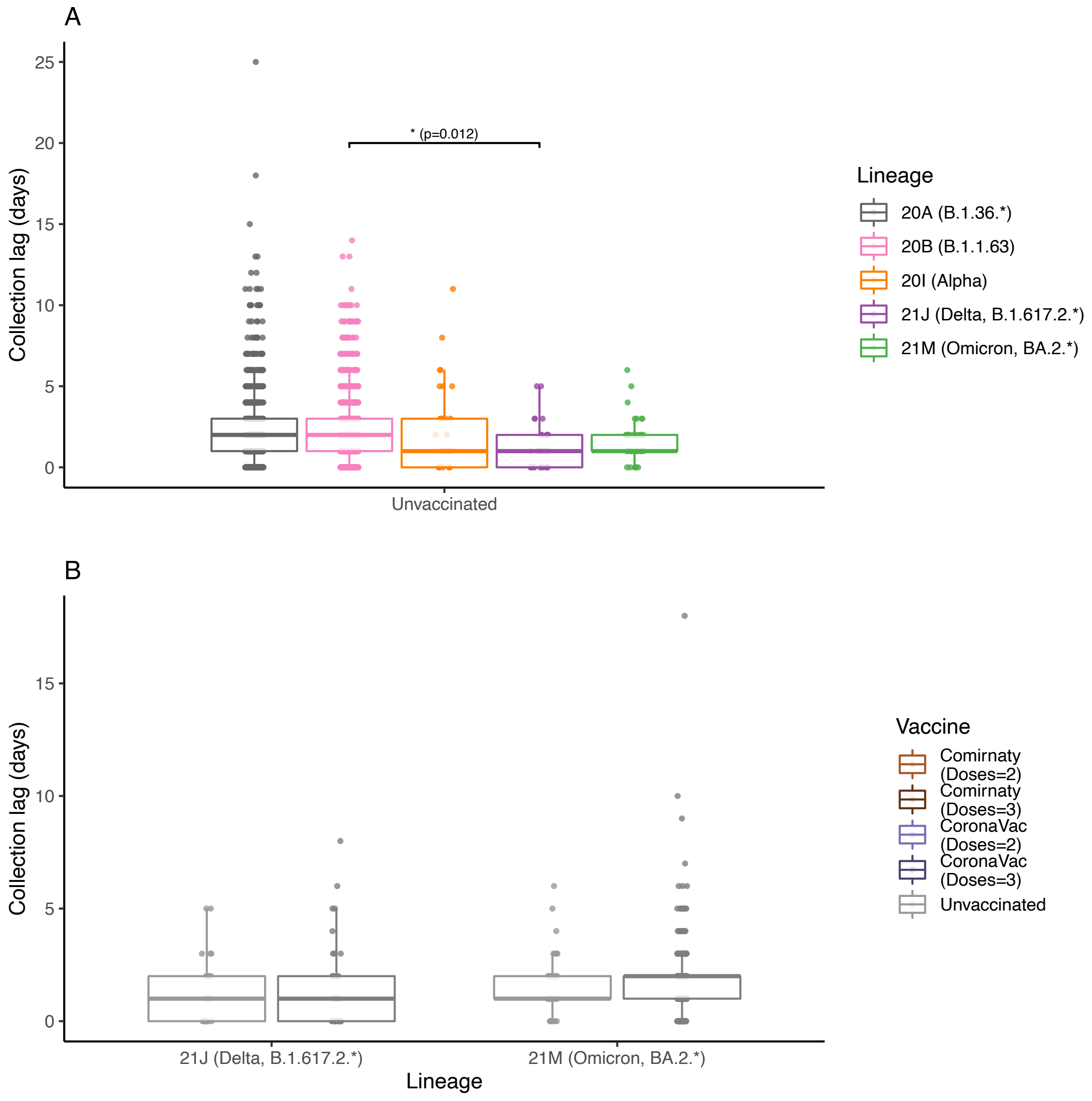

Supplementary Figure 13. Distribution of Ct values between different groups. (**A**) Distribution in unvaccinated samples; (**B**) Distribution in vaccinated samples. Pairwise comparisons within groups were tested by two-sided two-sample Wilcoxon tests; the pairs with BH adjusted P value ≤0.01 and ≤0.05 are labelled with "**" and "*" respectively. The number of biologically independent samples in each group are shown in Supplementary Table 5. For all box plots, the bold horizontal line inside the box shows the median, the upper and lower edges of the box indicate the first and the third quartiles, and whiskers extend to span a 1.5 interquartile range from the edges. Source data are provided as a Source Data file.

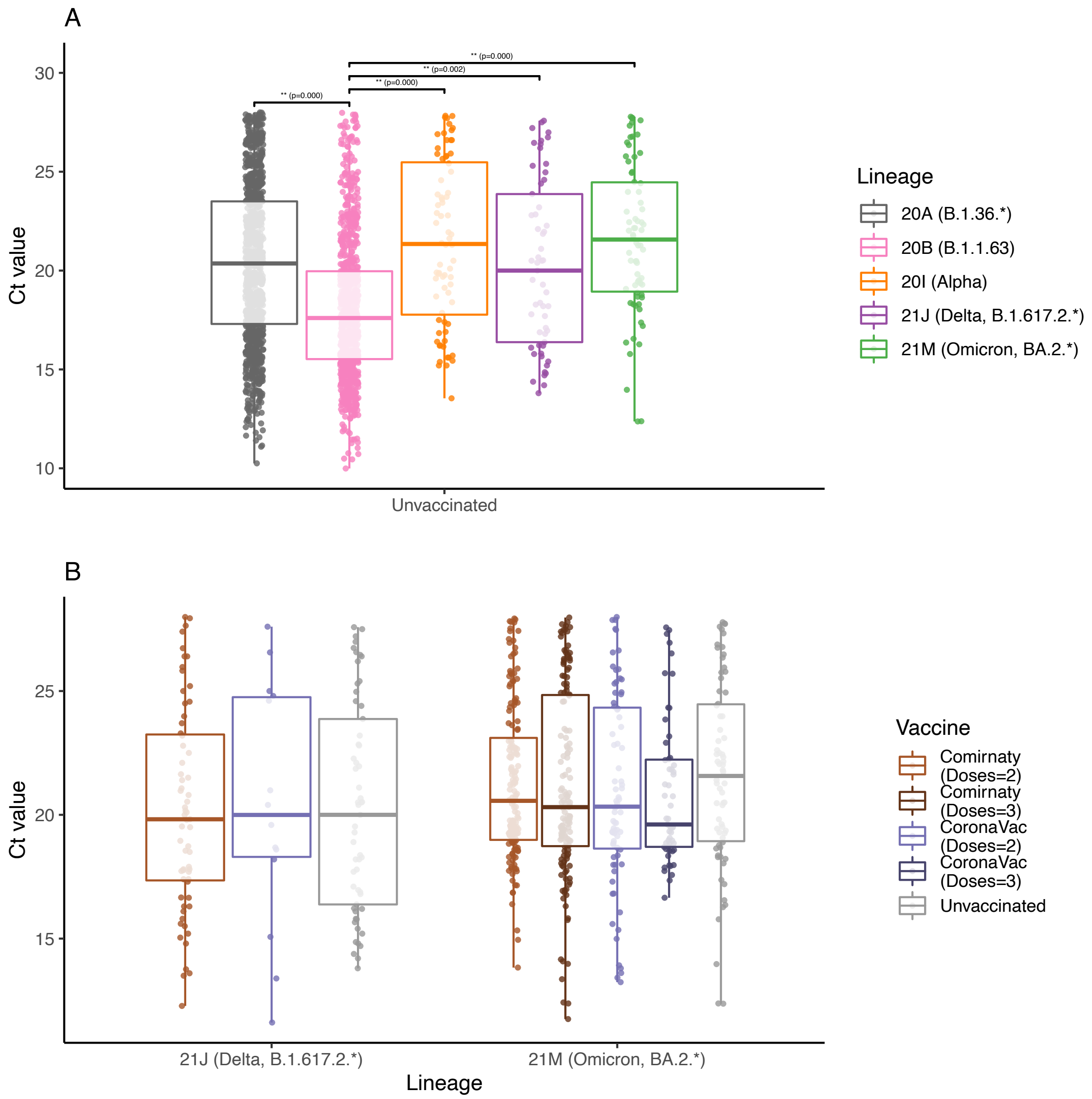

Supplementary Figure 14. Correlation between age and (A) Ct value, and (B) incidence of iSNVs. Statistical test used is two-sided Pearson correlation test with no adjustment for multiple comparisons. The error bands in grey show the 95% confidence interval for linear regression. Source data are provided as a Source Data file.

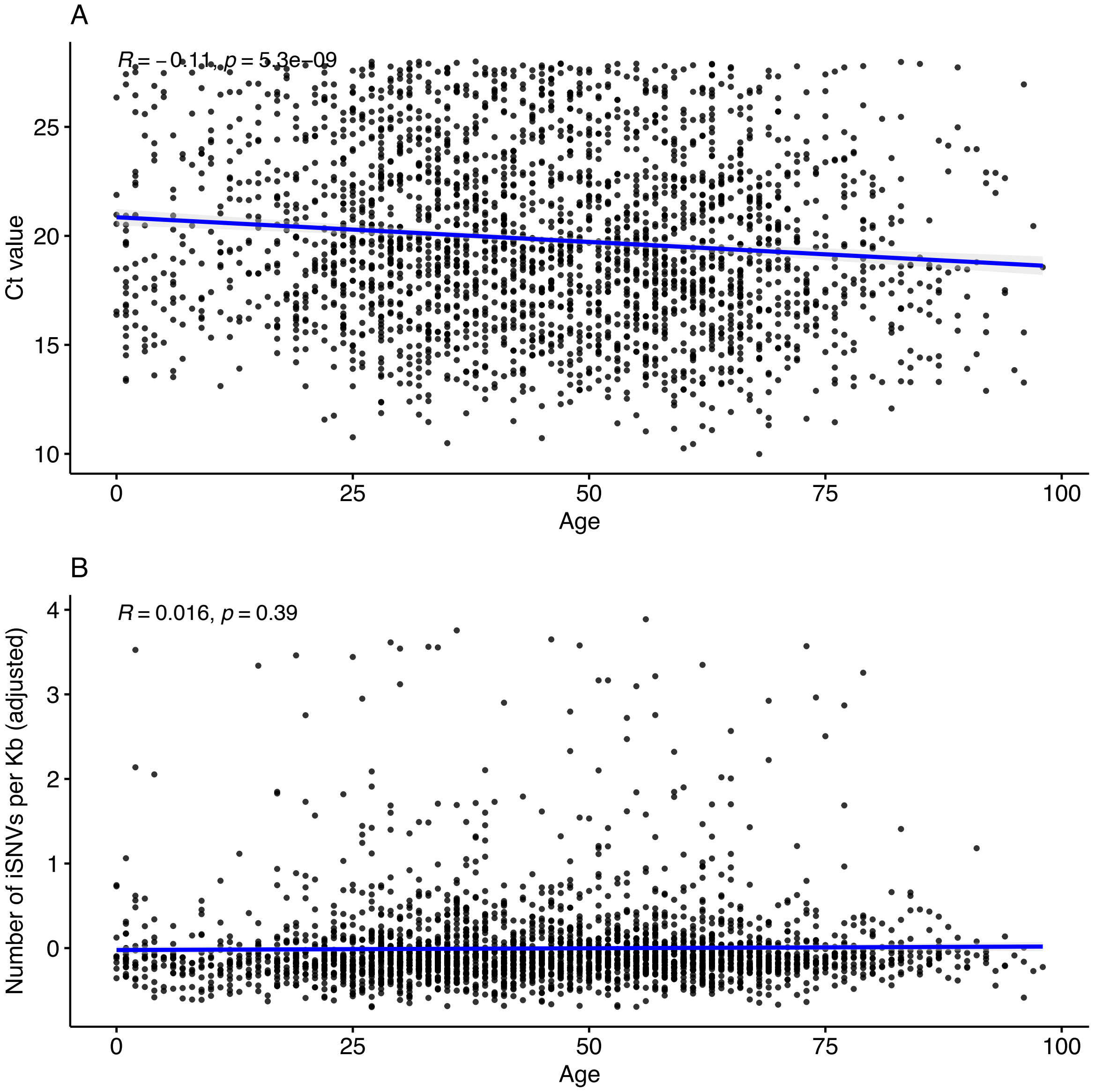

Supplementary Figure 15. Analysis of serial diluted samples. (**A**) sequencing depth; (**B**) genome coverage and average depth; (**C**) number of identified iSNVs using different thresholds. Source data are provided as a Source Data file.

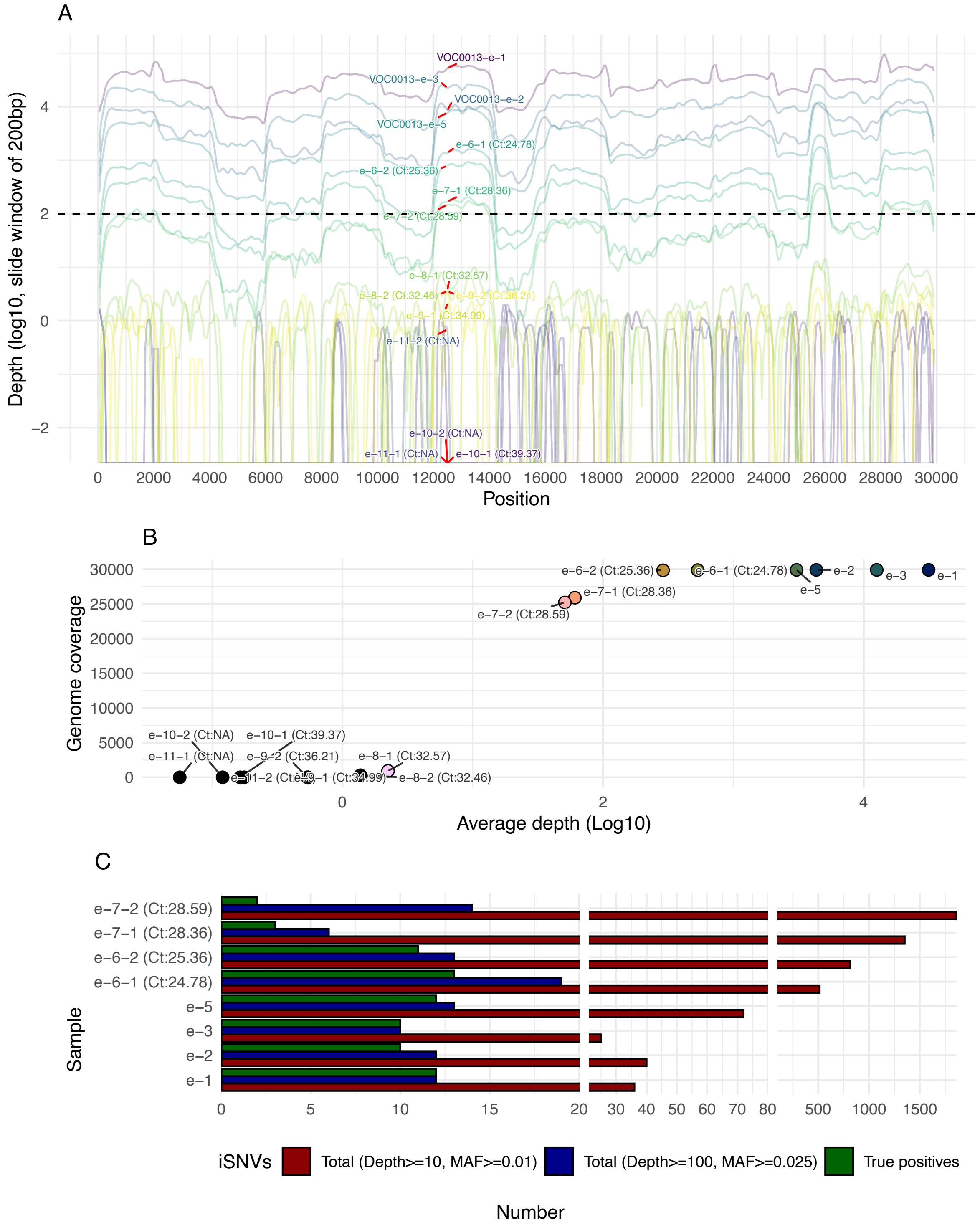

Supplementary Figure 16. Receiver operating characteristic (ROC) curves of different depth cut-offs in serial dilution samples. The dashed rectangles show the 95% confidence intervals (Reference: Pepe, M.S. "The Statistical Evaluation of Medical Tests for Classification and Prediction." Oxford (2003).) of true positive fraction and false positive fraction. **(A)** MAF thresholds of 1%; **(B)** MAF thresholds of 2%; **(C)** MAF thresholds of 3%; **(D)** MAF thresholds of 4%; Source data are provided as a Source Data file.

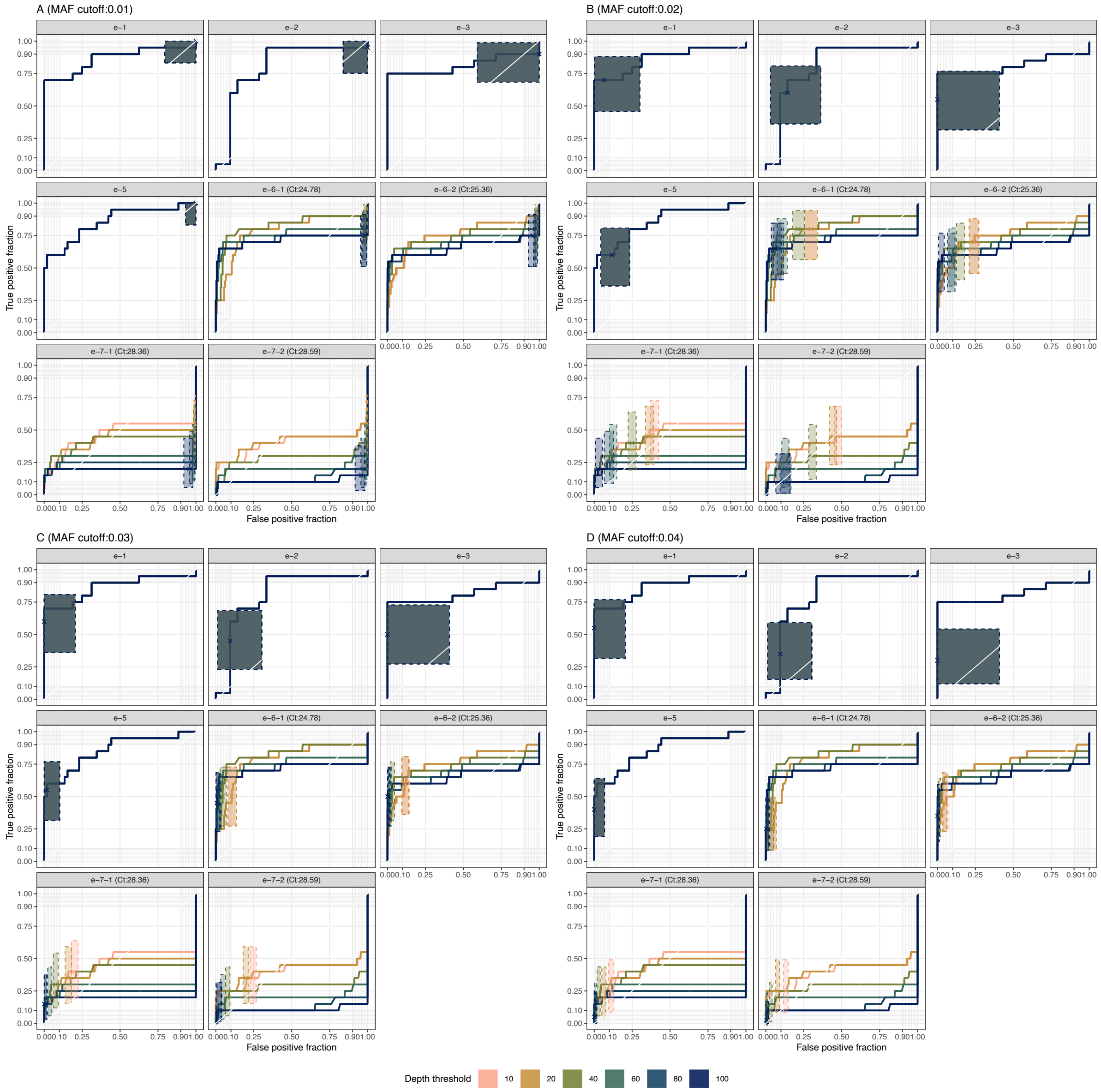

Supplement: Supplementary file 1 — Supplementary Information [file 41467_2023_37468_MOESM1_ESM.pdf]
