# Supplementary material for: Within-host genetic diversity of SARS-CoV-2 lineages in unvaccinated and vaccinated individuals
Source: Nat Commun. 2023 Mar 31;14:1793. doi: 10.1038/s41467-023-37468-y (PMC10063955; doi:10.1038/s41467-023-37468-y)
Supplement: Supplementary file 3 — Reporting Summary [file 41467_2023_37468_MOESM3_ESM.pdf]

nature portfolio

Corresponding author(s): Leo L.M. Poon

Last updated by author(s): 2023-02-23

Reporting Summary

Nature Portfolio wishes to improve the reproducibility of the work that we publish. This form provides structure for consistency and transparency in reporting. For further information on Nature Portfolio policies, see our Editorial Policies and the Editorial Policy Checklist.

Please do not complete any field with "not applicable" or n/a. Refer to the help text for what text to use if an item is not relevant to your study.
For final submission: please carefully check your responses for accuracy; you will not be able to make changes later.

Statistics

For all statistical analyses, confirm that the following items are present in the figure legend, table legend, main text, or Methods section.

n/a	Confirmed	
☐	☒	The exact sample size (*n*) for each experimental group/condition, given as a discrete number and unit of measurement
☐	☒	A statement on whether measurements were taken from distinct samples or whether the same sample was measured repeatedly
☐	☒	The statistical test(s) used AND whether they are one- or two-sided<br>*Only common tests should be described solely by name; describe more complex techniques in the Methods section.*
☐	☒	A description of all covariates tested
☐	☒	A description of any assumptions or corrections, such as tests of normality and adjustment for multiple comparisons
☐	☒	A full description of the statistical parameters including central tendency (e.g. means) or other basic estimates (e.g. regression coefficient) AND variation (e.g. standard deviation) or associated estimates of uncertainty (e.g. confidence intervals)
☐	☒	For null hypothesis testing, the test statistic (e.g. *F*, *t*, *r*) with confidence intervals, effect sizes, degrees of freedom and *P* value noted<br>*Give P values as exact values whenever suitable.*
☒	☐	For Bayesian analysis, information on the choice of priors and Markov chain Monte Carlo settings
☒	☐	For hierarchical and complex designs, identification of the appropriate level for tests and full reporting of outcomes
☒	☐	Estimates of effect sizes (e.g. Cohen's *d*, Pearson's *r*), indicating how they were calculated

Our web collection on statistics for biologists contains articles on many of the points above.

Software and code

Policy information about availability of computer code

Data collection	No software was used for data collection in this study.
Data analysis	For NGS data analyses: BWA-MEM2 (v2.0pre2), iVar (v1.3.1) , SNPGenie (v1.0), samtools (v1.1), pysamstats (v1.1.2).<br>For multiple other analysis: R (v4.1.0 and v4.1.3) with custom codes (https://github.com/Leo-Poon-Lab/mutations-under-sarscov2-vaccination).

For manuscripts utilizing custom algorithms or software that are central to the research but not yet described in published literature, software must be made available to editors and reviewers. We strongly encourage code deposition in a community repository (e.g. GitHub). See the Nature Portfolio guidelines for submitting code & software for further information.

Data

Policy information about availability of data

All manuscripts must include a data availability statement. This statement should provide the following information, where applicable:
- Accession codes, unique identifiers, or web links for publicly available datasets
- A description of any restrictions on data availability
- For clinical datasets or third party data, please ensure that the statement adheres to our policy

The sequencing data generated in this study have been deposited in the NCBI Sequence Read Archive (SRA) under Bioproject accession code PRJNA930974 (https://www.ncbi.nlm.nih.gov/bioproject/?term=PRJNA930974). The processed anonymised metadata are deposited at Github (https://github.com/Leo-Poon-Lab/mutations-under-sarscov2-vaccination/blob/main/metadata/df_samples_anonymised.csv). SARS-CoV-2 reference genome (Wuhan-Hu-1, GenBank: MN908947.3) is available on GenBank. The SARS-CoV-2 CD8+ and CD4+ T cell epitope data were retrieved from the dashboard reported by us (https://www.mckayspcb.com/SARS2TcellEpitopes/; accessed on 15 November 2022) and the Immune Epitope Database (IEDB) (https://www.iedb.org; accessed on 15 November 2022). Source data are provided with this paper.

Human research participants

Policy information about studies involving human research participants and Sex and Gender in Research.

Reporting on sex and gender	The sex and gender of the hosts are disclosed in the anonymised metadata.
Population characteristics	All samples were collected from COVID-19 patients detected in Hong Kong. The dataset includes samples from 2,820 individuals, where 1400 are male, 1413 are female and 7 are not available. The mean and median age of the studied population are 45.23 and 45 respectively.
Recruitment	No patient was recruited in this study. These were archived SARS-CoV-2 samples confirmed by citywide public health screening programs.
Ethics oversight	This study was conducted under ethical approval from the Institutional Review Board of the University of Hong Kong (UW 20-168).

Note that full information on the approval of the study protocol must also be provided in the manuscript.

Field-specific reporting

Please select the one below that is the best fit for your research. If you are not sure, read the appropriate sections before making your selection.

☒ Life sciences ☐ Behavioural & social sciences ☐ Ecological, evolutionary & environmental sciences

Life sciences study design

All studies must disclose on these points even when the disclosure is negative.

Sample size	This is a genomic study that used all available genomic data (N=2820) to infer the within-host diversity of SARS-CoV-2.
Data exclusions	The samples and data with insufficient quality (see Methods) were excluded from the analysis.
Replication	Amongst the manually generated serially-diluted samples, 10 samples (from e-6 to e-11) which with Ct values >24 were sequenced in duplicates. All attempts at replication were successful. This study does not involve other biological experiments and replication were not applicable for the other analysis.
Randomization	Randomization was not applicable as in this study we tried to use all available data from routine surveillance.
Blinding	Blinding was not relevant as routine surveillance samples with de-identified patient data were utilised in the study.

Reporting for specific materials, systems and methods

We require information from authors about some types of materials, experimental systems and methods used in many studies. Here, indicate whether each material, system or method listed is relevant to your study. If you are not sure if a list item applies to your research, read the appropriate section before selecting a response.

Materials & experimental systems

n/a	Involved in the study
☐	☐ Antibodies
☐	☐ Eukaryotic cell lines
☐	☐ Palaeontology and archaeology
☐	☐ Animals and other organisms
☐	☐ Clinical data
☐	☐ Dual use research of concern

Methods

n/a	Involved in the study
	☐ ChIP-seq
	☐ Flow cytometry
	☐ MRI-based neuroimaging

This checklist template is licensed under a Creative Commons Attribution 4.0 International License, which permits use, sharing, adaptation, distribution and reproduction in any medium or format, as long as you give appropriate credit to the original author(s) and the source, provide a link to the Creative Commons license, and indicate if changes were made. The images or other third party material in this article are included in the article's Creative Commons license, unless indicated otherwise in a credit line to the material. If material is not included in the article's Creative Commons license and your intended use is not permitted by statutory regulation or exceeds the permitted use, you will need to obtain permission directly from the copyright holder. To view a copy of this license, visit http://creativecommons.org/licenses/by/4.0/
